# The single-cell transcriptional landscape of lung cells from PCV2d-infected mice

**DOI:** 10.3389/fmicb.2025.1554961

**Published:** 2025-03-24

**Authors:** Yunlong Chen, Gang Fan, Bin Yang, Xinyi Fan, Haiyan Chen, Zhuoyuan Ma, Jiao Lou, Jingmei Xu, Yan Wang, Shiqiang Zhang

**Affiliations:** ^1^College of Veterinary Medicine/Shaanxi Stem Cell Engineering Research Center, Northwest A&F University, Yangling, China; ^2^Key Laboratory of Livestock Biology, Northwest A&F University, Yangling, China

**Keywords:** mice, single-cell RNA sequencing, PCV2d, host antiviral response, circovirus, lung tissue

## Abstract

**Introduction:**

Porcine Circovirus (PCV2) infection is prevalent in pig farming and causes significant economic losses. In recent years, the PCV2d subtype has become the most prevalent genotype worldwide, exhibiting higher virulence, leading to more severe viremia and organ damage. Therefore, studying the biological characteristics of the PCV2d subtype is of great significance.

**Methods:**

We established a PCV2d infection model using BALB/c mice and employed single-cell RNA sequencing (scRNA-seq) to systematically analyze the transcriptome of 10 cell types in the lung tissues of infected mice. We developed a comprehensive marker gene catalog for these cell types.

**Results:**

Compared to uninfected mice, PCV2d infection induced extensive viral replication and immunosuppressive responses in most cell types. Monocyte macrophages with high levels of viral replication, pro-inflammatory cytokines, and various cell population interactions occurring through CD40-CD40L and CXCL14-CXCR4 were identified. These cells predominantly mediate antigen presentation and processing pathways *in vivo*, contributing to PCV2d-driven inflammatory lung injury.

**Discussion:**

Our data uncovered a complex unique immune response scenario in the lung tissue of mice after PCV2d infection, deciphering the potential mechanisms underlying PCV2d-driven inflammatory responses in mice. Furthermore, this study provides a rich database for the molecular basis of different cell types' responses to PCV2d infection.

## Introduction

PCV2 is ubiquitous and a major pathogen of PCV2-associated disease (Porcine Circovirus Associated Disease, PCVAD), leading to severe economic losses in the swine industry (Rose et al., 2012; Meng, 2013). PCV2 is classified into five genotypes (PCV2a–PCV2e) based on its open reading frame (ORF) 2 sequence. PCV2a was the predominant genotype globally until 2000, while a genotypic shift to PCV2b occurred in 2003 (Dupont et al., 2008; Opriessnig et al., 2014); PCV2c was discovered in Denmark (Franzo et al., 2015). In 2010, an emerging PCV2d genotype called mutant PCV2b (mPCV2b) was identified in PMWS pigs in China (Guo et al., 2010), and PCV2e was found in the United States and Mexico (Harmon et al., 2015; Davies et al., 2016). Currently, the PCV2d genotype has become the most popular genotype in the U.S. (Wang et al., 2020), China (Hou et al., 2019), Russia (Raev et al., 2021), Italy (Dei Giudici et al., 2019) and South Korea (Kwon et al., 2017).

In a previous study, tissue and serum samples collected from diseased or seemingly healthy wild boars in 19 regions of China between 2018 and 2020 were examined for the prevalence of PCV2 infection, with a PCV2d infection rate of 52.5% (Gong et al., 2023), this suggests that PCV2d is the main prevalent subtype of PCV2. Therefore, there is a need for a deeper understanding of the determinants and pathogenesis of PCV2d infection in order to develop effective methods of control. Despite the informative biomarker profiles identified to date, technical limitations have hindered a holistic view of the immune cellular processes underlying immune setpoints that predict and potentially determine optimal responses (Germain and Schwartzberg, 2011; Germain, 2018). Large numbers of blood transcriptome profiles are disturbed due to large interindividual variation in circulating immune cell subpopulation frequencies (Tsang et al., 2014; Roederer et al., 2015; Lu et al., 2016). In recent years, high-throughput single-cell RNA sequencing technology (scRNA-seq) has been widely used to gain a comprehensive understanding of cellular responses and transcriptional profiles in various cellular or animal tissues (Madissoon et al., 2019). In particular, scRNA-seq technology has further deepened our understanding of cellular composition, single-cell transcriptional landscapes, intercellular communication, and cellular states within tissues (Wu et al., 2017; Liu et al., 2020; Zhang et al., 2020).

Replication of PCV2 in experimentally inoculated 6-week-old BALB/c mice has been demonstrated (Kiupel et al., 2001). In mice, PCV2 induced pathological changes similar to those seen in pigs infected with PCV2, manifesting as depletion of lymphoid tissue cells, granulomatous inflammation, and so on. In this study, we infected mice with PCV2d virus and performed single-cell sequencing analysis. Through this high-throughput technical means, we successfully obtained transcriptomic information of single cells in infected mice. The results showed that PCV2d virus infection induced a wide range of changes in the transcriptome of cells in mice, especially significant alterations in the expression of genes related to immune response and cellular stress response. These findings provide an important molecular basis for our in-depth understanding of the pathogenic mechanism of PCV2d virus and the host response to the virus, and offer new clues for subsequent drug development and disease treatment strategies.

## Results

### Single-cell sequencing analysis process

**Figure 1:**
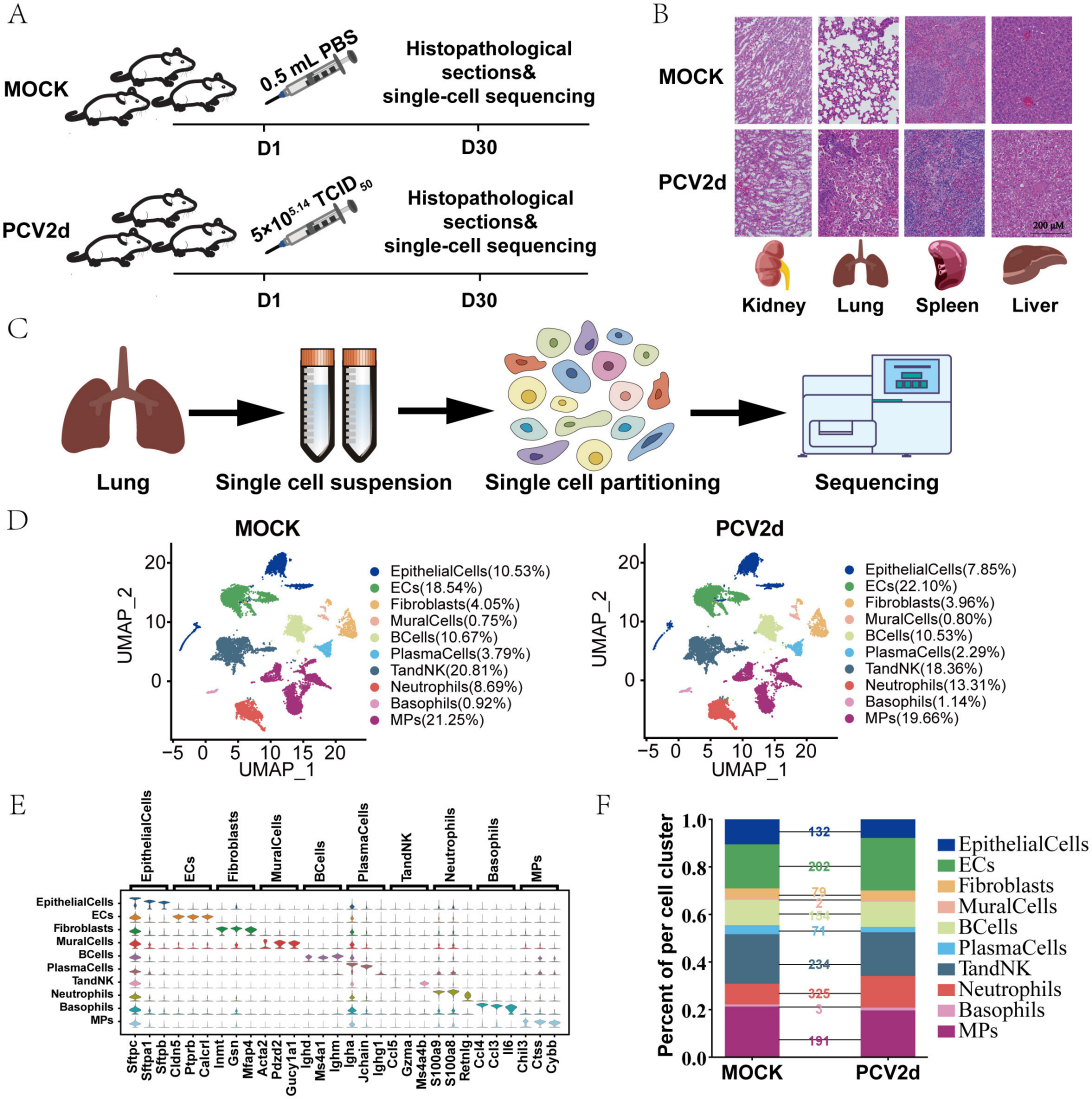
Design and differences in lung cell composition studied throughout PCV2d infection of BALB/c mice. **(A)** Schematic diagram of the study design. This study was done for two groups of BALB/c mice with different treatments. **(B)** On the 30th day, the liver, spleen, lungs and kidneys of mice were taken for pathological section observation. **(C)** The lung tissue with the most pronounced differences after PCV2d infection was taken on day 30 for single-cell sequencing. **(D)** Overview of cell populations in the integrated single-cell transcriptome. Each dot corresponds to a cell, colored according to cell type. MOCK: injected with PBS; PCV2d: injected with PCV2d. **(E)** Expression levels of cell typing genes in cell type clusters. Boxplot plots represent the expression distribution of selected typical cell markers in 10 cell clusters. Columns represent selected marker genes and rows represent clusters. **(F)** The percentage of each of the 10 cellular subpopulations in the MOCK group, PCV2d group and the number of differential genes in each subpopulation were counted.

To investigate the pathogenesis and mechanism of PCV2d infections in mice, lung samples were collected from Mock-infected mice (*n* = 3) and PCV2d-infected mice (*n* = 3). The lung samples were collected on day 30 (Figure 1A). After filtering out cells with low quality, we obtained transcriptome datasets from 12,517 cells for Mock group and 16,652 cells for PCV2d-infected group. BALB/c mice were grouped into groups of three mice each, with males and females randomized. The flow chart of the experimental design (Figure 1A) demonstrated that 5 × 10^5.14^ TCID_50_ of PCV2d virus (viral titer of 10^5.14^ TCID_50_/0.1mL) and 0.5 mL of PBS were injected on the first day, and pathology sections and single-cell sequencing analyses were done on the 30th day for the two groups of mice, respectively. Pathological section examination was done on the liver, spleen, lung and kidney of the two groups of mice (Figure 1B), in which the lung tissues were significantly different after PCV2d infection, showing lung fibrosis and typical inflammatory reaction. Therefore, the lung tissues were extracted and sent to Singleron Biotechnologies for sequencing (Figure 1C). Using genetic annotation of different cells provided by the company, the cell samples were categorized into 10 cell types (Figure 1D), including *Epithelial cells* (*EpithelialCells*, 10.53% in the MOCK group and 7.85% in the PCV2d group), *Endothelial cells* (*ECs*, 18.54% in the MOCK group and 22.10% in the PCV2d group), *Fibroblasts* (4.05% in the MOCK group and 3.96% in the PCV2d group), *Mural cells* (*MuralCells*, 0.75% in the MOCK group and 0.80% in the PCV2d group), *B cells* (*BCells*, 10.67% in the MOCK group and 10.53% in the PCV2d group), *Plasma cells* (*PlasmaCells*, 3.79% in the MOCK group and 2.29% in the PCV2d group), *T and NK cells* (*TandNK*, 20.81% in the MOCK group and 18.36% in the PCV2d group), *Neutrophils* (8.69% in the MOCK group and 13.31% in the PCV2d group), *Basophils* (0.92% in the MOCK group and 1.14% in the PCV2d group), *Mononuclear phagocytes* (*MPs*, 21.25% in the MOCK group and 19.66% in the PCV2d group), as well as demonstrating the downscaled clustering of 10 cell types by specific marker genes (Figure 1E), the top three specific marker genes for *Epithelialcells* are Sftpc, Sftpa1, and Sftpb, for *ECs* are Cldn5, Ptprb and Calcrl, for *Fibroblasts* are Inmt, Gsn and Mfap4, for *Muralcells* are Acta2, Pdzd2 and Gucy1a1, for *BCells* are Ighd, Ms4a1 and Ighm, for *PlasmaCells* are Igha, Jchain and Ighg1, for *TandNK* are Ccl5, Gzma and Ms4a4b, for *Neutrophils* are S100a9, S100a8 and Retnlg, for *Basophils* are Ccl4, Ccl3 and Il6, and for MPs are Chil3, Ctss and Cybb. Percentage of cells in each subpopulation and the number of differential genes in each subpopulation were counted for both groups of mice (Figure 1F). The results showed that after PCV2d infection in mice, the most obvious subpopulation differences were caused by *Neutrophils, TandNK, ECs, MPs, BCells, EpithelialCells* and *Fibroblasts*, respectively.

### PCV2d infection leads to abnormalities in antigen processing and presentation pathway in MPs

**Figure 2:**
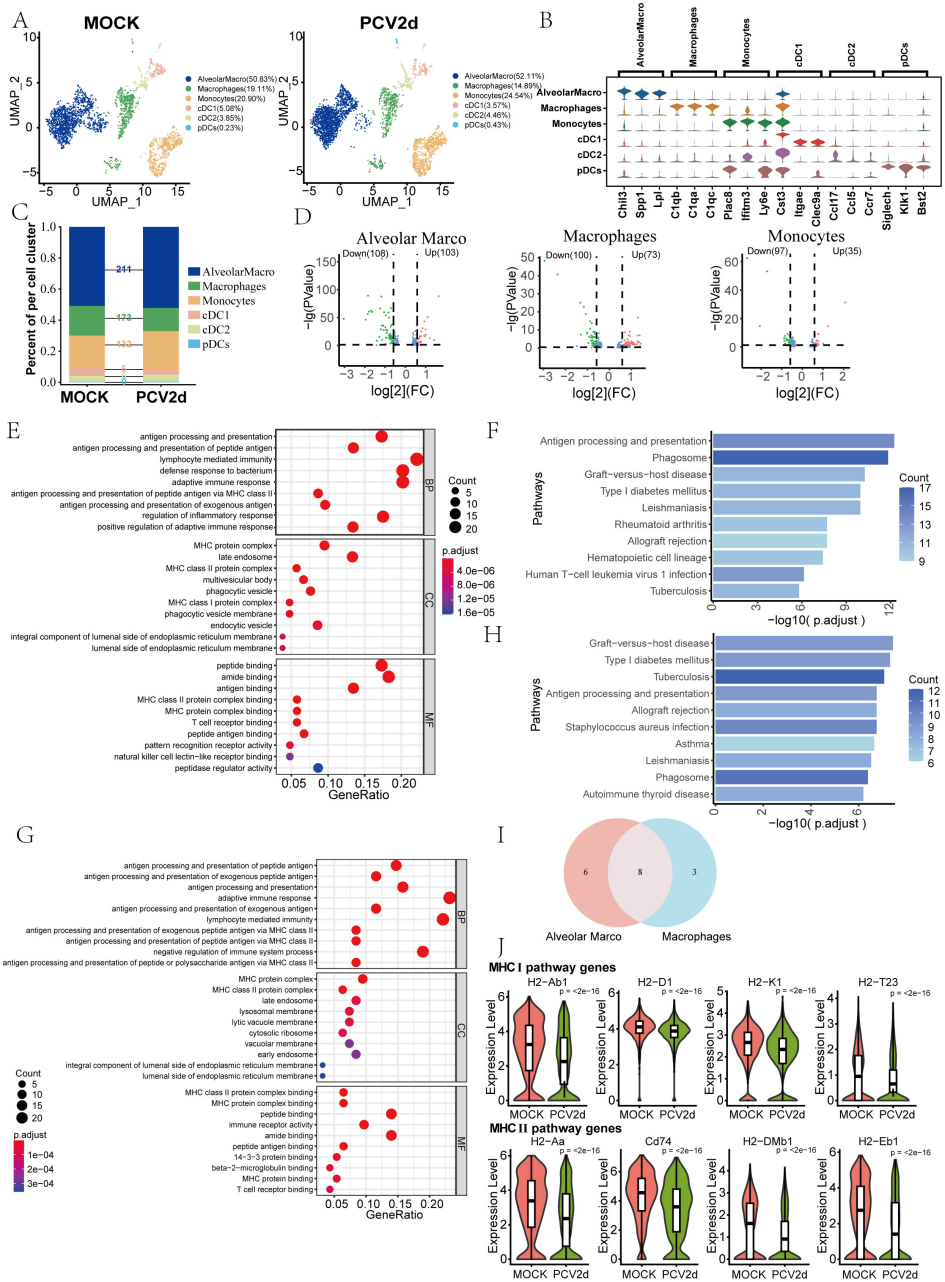
Differential changes in *MPs* under the two treatment groups. **(A)** Reduced-dimensional grouping of MOCK and PCV2d group MPs. **(B)** Six subpopulation subgroups specific to the first three marker genes. **(C)** Occupancy stacking diagrams and differential gene counts for the six subpopulations. **(D)** Volcano maps of three groups of up- and down-regulated genes with large differences. **(E)** GO enrichment results for *Alveolar Marco* down-regulated genes. **(F)** KEGG enrichment results of *Alveolar Marco* down-regulated genes. **(G)** GO enrichment results for *Macrophages* down-regulated genes. **(H)** KEGG enrichment results for *Macrophages* down-regulated genes. **(I)** Venn Analysis of Genes in the Antigen Presentation and Processing Pathway Enriched by *Alveolar Marco* and *Macrophages* Downregulated Genes. **(J)** The eight common genes are shown separately according to the MHC I and MHC II pathways. Statistical significance of measurements was assessed using unpaired two-tailed *T*-tests.

Macrophages exist as a heterogeneous population of cells in the lungs and various fluid compartments, where they act as sentinels to detect and respond to infections and aid in adaptive immune responses through antigen presentation to T cells (Cline et al., 2017). Based on the results of this annotation, *MPs* were categorized into six major subgroups (Figure 2A), including *Alveolar macrophages* (*AlveolarMacro*, 50.83% in the MOCK group and 52.11% in the PCV2d group), *Macrophages* (19.11% in the MOCK group and 14.89% in the PCV2d group), *Monocytes* (20.90% in the MOCK group and 24.54% in the PCV2d group), *Conventional type 1 dendritic cells* (*cDC1*, 5.08% in the MOCK group and 3.57% in the PCV2d group), *Conventional type 2 dendritic cells* (*cDC2*, 3.85% in the MOCK group and 4.46% in the PCV2d group), *Plasmacytoid dendritic cells* (*pDCs*, 0.23% in the MOCK group and 0.43% in the PCV2d group). The descending subpopulation-specific marker genes for each subpopulation of cells are shown (Figure 2B), the top three specific marker genes for *AlveolarMacro* are Chil3, Spp1 and Lpl, for Macrophages are C1qb, C1qa and C1qc, for *Monocytes* are Plac8, Ifitm3 and Ly6e, for *cDC1* are Cst3, Itgae and Clec9a, for *cDC2* are Ccl17, Ccl5 and Ccr7, and for *pDCs* are Siglech, Klk1 and Bst2. We further performed statistical analysis on the differential genes of the six subpopulations (Figure 2C), and the results showed that the three subpopulations with the largest differences were *AlveolarMacro, Macrophages*, and *Monocytes*. Volcano plots of the three subpopulations of differential genes were analyzed (Figure 2D). Since the down-regulated genes of the three subgroups accounted for a larger proportion, Gene Ontology (GO) and Kyoto Encyclopedia of Genes and Genomes (KEGG) enrichment was performed for the down-regulated genes of *AlveolarMacro* and *Macrophages*, respectively (Figures 2E–H), and GO and KEGG enrichment for the up-regulated genes was shown (Supplementary Figures S1A–D). The genes in the antigen processing and presentation pathway of the two subgroups were analyzed by Venn analysis (Figure 2I). Venn analysis was performed for six genes specific to *AlveolarMacro*, namely H2-Q7, Ctss, B2m, Ctsl, Lgmn and Tnf, and three genes specific to *Macrophages*, namely H2-DMa, Hspa1b and Cd4, whose boxplot Expression is shown (Supplementary Figure S1E). There are eight genes in both subpopulations in the antigen processing and presentation pathway, grouped according to the functional roles assumed in MHC I and MHC II, respectively, and the gene boxplot expression is shown (Figure 2J). The activation of monocyte macrophages promotes an inflammatory response, which may lead to lung tissue damage during viral infections. They affect the local microenvironment at the site of infection by releasing inflammatory mediators, which influence viral clearance and tissue repair (Segalés, 2012).

### Differential genes in MPs function in antigen processing and presentation pathway

**Figure 3:**
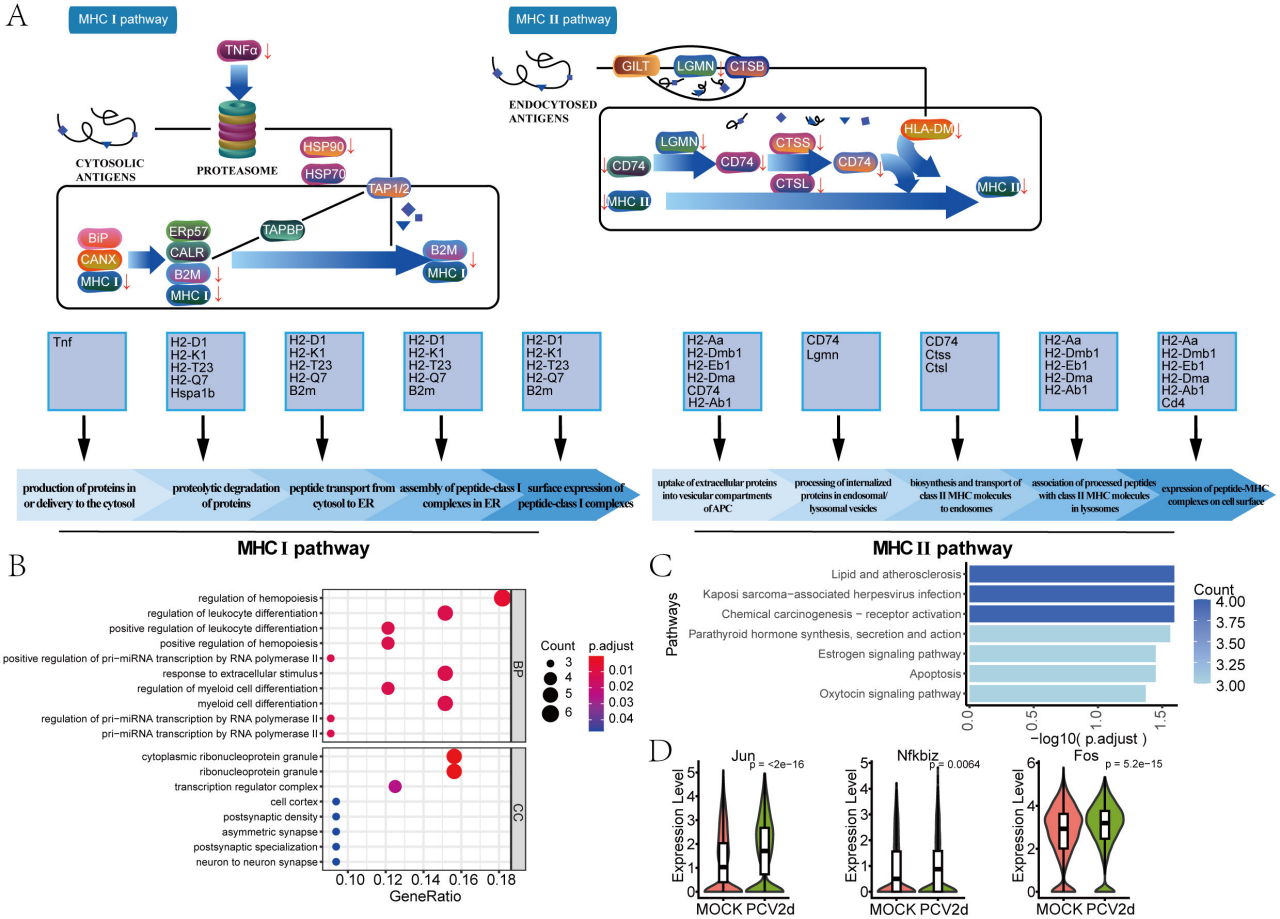
*MPs* antigen presentation processing pathway gene functioning link and monocytes subpopulation differential gene enrichment. **(A)** Genes associated with the antigen presentation processing pathway assume roles in two different presentation modes of linkage. **(B)** GO enrichment results for *Monocytes* up-regulated genes. **(C)** KEGG enrichment results of *Monocytes* up-regulated genes. **(D)** Boxplot expression of five genes in the leukocyte differentiation pathway. Statistical significance of measurements was assessed using unpaired two-tailed *T*-tests.

We analyzed the specific genes in the antigen processing and presentation pathway that are dysregulated following PCV2d infection. Their specific roles in the two antigen presentation processes are shown (Figure 3A). We conclude that after PCV2d infection, all of the above genes are abnormal at different points in the antigen processing and presentation pathway, the most important of which are polypeptide transport in MHC I and polypeptide processing in MHC II, leading to immune escape of the virus. We further analyzed the GO and KEGG enrichment of up-regulated genes in the subpopulation of Monocytes (Figures 3B, C), and the GO and KEGG enrichment of down-regulated genes are shown (Supplementary Figures S1F, G). We extracted the three genes on the leukocyte differentiation pathway in the subpopulation after PCV2d infection boxplot expression (Figure 3D), and the results showed that all of them were elevated to different degrees. We then did a cellular mimetic time-series analysis of the three subpopulations with the highest variance. The proposed chronological analysis of the three subpopulations under different treatment groups is shown (Supplementary Figure S1H). The results showed abnormalities in *Alveolarmacro* differentiation toward *Macrophage* and *Monocytes*, which were also associated with immune escape from PCV2d. The network diagram of PAGA proposed chronological analysis and the corresponding UMAP are shown (Supplementary Figure S2A); this also reflects the relationship between the three subgroups of differentiation in the body, from *Monocytes* to *AlveolarMacro*, and finally to *Macrophages*. The lines (solid and dashed) represent the strength of the differentiation relationship.

### PCV2d infection leads to abnormalities in T cell activation and differentiation pathway in T and NK cells

**Figure 4:**
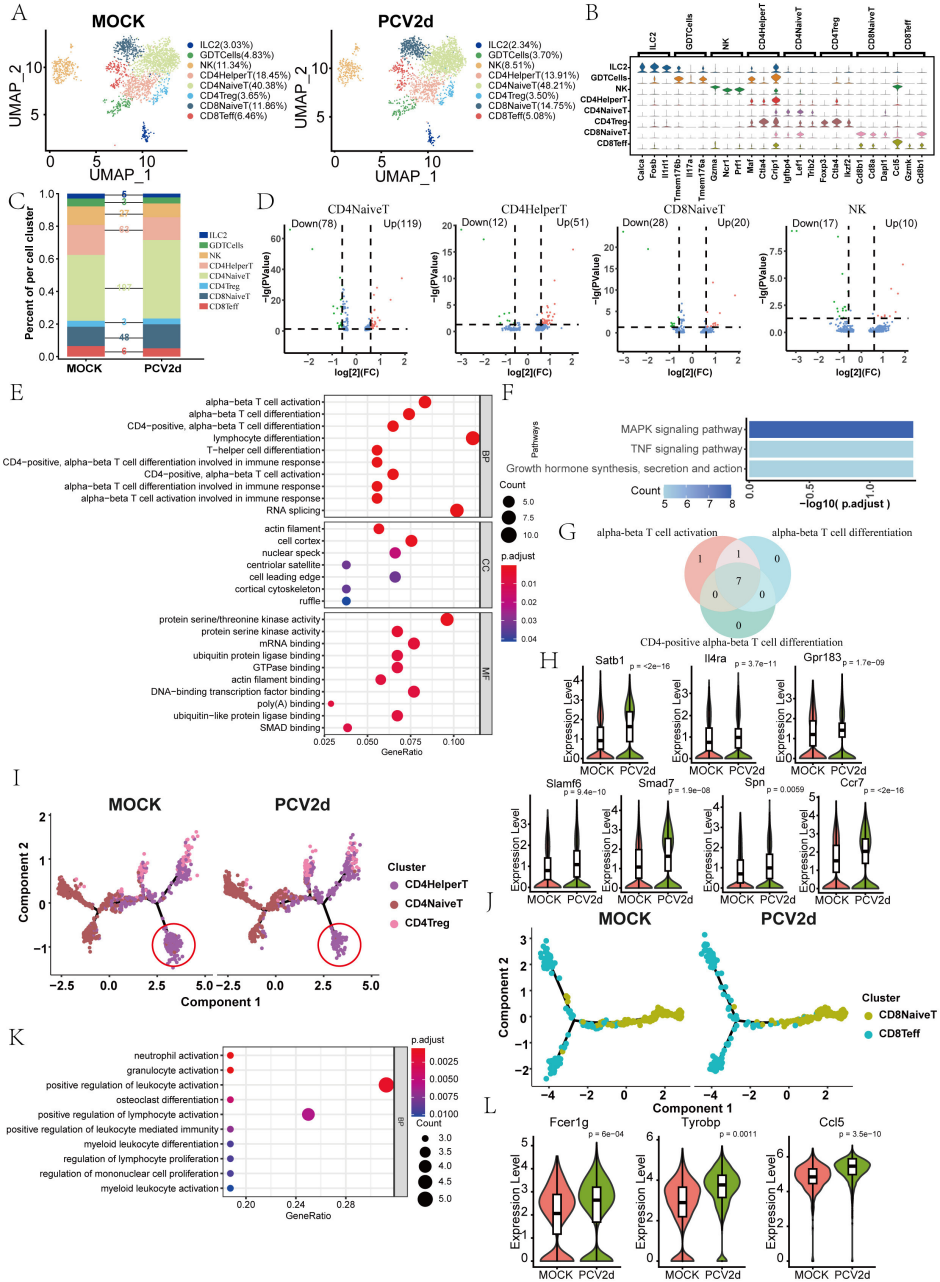
Differential changes in *TandNK* after infection by PCV2d. **(A)** Reduced-dimensional binning of *TandNK* for MOCK and PCV2d groups. **(B)** Eight subpopulation subgroup-specific top three Marker genes. **(C)** Occupancy stacking diagrams and differential gene counts for eight subpopulations. **(D)** Volcano maps of statistically up- and down-regulated genes for the four subgroups with high variance. **(E)** GO enrichment results for genes upregulated by *CD4NaiveT* cells. **(F)** KEGG enrichment results for genes upregulated by *CD4NaiveT* cells. **(G)** Venn analysis of T cell activation and differentiation pathway genes. **(H)** Boxplot expression of seven common genes. Statistical significance of measurements was assessed using unpaired two-tailed *T*-tests. **(I)** *CD4NaiveT* to *CD4HelperT* and *CD4treg* proposed temporal differentiation, in which there was a significant decrease in the degree of differential differentiation at the end of *CD4HelperT* in the PCV2d-treated group. **(J)** *CD8NaiveT* cells to *CD8Teff* mimetic temporal differentiation. **(K)** KEGG enrichment results for genes down-regulated by *NK cells*. **(L)** Boxplot expression of three genes of the neutrophil activation pathway. Statistical significance of measurements was assessed using unpaired two-tailed *T*-tests.

Secondary clustering of the mouse T and NK major classes was performed, and subdivision annotation yielded eight different isoforms (Figure 4A), including *Group 2 innate lymphoid cells* (*ILC2*, 3.03% in the MOCK group and 2.34% in the PCV2d group), *Gamma delta T cells* (*GDTCells*, 4.83% in the MOCK group and 3.70% in the PCV2d group), *Natural killer cells* (*NK*, 11.34% in the MOCK group and 8.51% in the PCV2d group), *CD4*^+^ *T helper cells* (*CD4HelperT*, 18.45% in the MOCK group and 13.91% in the PCV2d group), *CD4*^+^ *naive T cells* (*CD4NaiveT*, 40.38% in the MOCK group and 48.21% in the PCV2d group), *CD4*^+^ *regulatory T cells* (*CD4Treg*, 3.65% in the MOCK group and 3.50% in the PCV2d group), *CD8*^+^ *naive T cells* (*CD8NaiveT*, 11.86% in the MOCK group and 14.75% in the PCV2d group), *CD8*^+^ *effector T cells* (*CD8Teff* , 6.46% in the MOCK group and 5.08% in the PCV2d group). We show that in each subpopulation of specific marker genes (Figure 4B), the top three specific marker genes for *Ilc2* are Calca, Fosb and Il1rl1, for *GDTCells* are Tmem176b, Il17a and Tmem176a, for *NK* are Gzma, Ncr1 and Prf1, for *CD4HelperT* are Maf, Ctla4 and Crip1, for *CD4NaiveT* are Igfbp4, Lef1 and Trib2, for *CD4Treg* are Foxp3, Ctla4 and Ikzf2, for *CD8NaiveT* are Cd8b1, Cd8a and Dapl1, for *CD8Teff* are Ccl5, Gzmk and Cd8b1. The percentage and differential genes of each subpopulation of cells infected by PCV2d were counted (Figure 4C), and the results showed that the number of differential genes was higher for *CD4NaiveT, CD4HelperT, CD8NaiveT* and *NK cells*. The gene volcano maps of these four subpopulations of cells were counted separately (Figure 4D). Since the *CD4NaiveT* cell subpopulation had the highest number of differential genes and a larger proportion of up-regulated genes, we therefore enriched for these differential genes, GO (Figure 4E) and KEGG (Figure 4F) enrichment of up-regulated genes were counted in *CD4NaiveT*, and GO and KEGG enrichment of down-regulated genes were also done (Supplementary Figures S2B, C). Enrichment to the immune-related T-cell differentiation pathway, and in the down-regulated GO and KEGG enrichment of the three subsets of *CD4HelperT, CD8NaiveT* and *NK* were similarly enriched to T cell differentiation pathways (Supplementary Figures S2D–I). Venn analysis of the three T cell activation and differentiation pathways (Figure 4G) showed that there were seven genes common to the three pathways, they are Satb1, Il4ra, Gpr183, Slamf6, Smad7, Spn and Ccr7 genes, and boxplot expression was extracted for these seven genes (Figure 4H). We next analyzed simulated temporal sequencing of the *CD4NaiveT* subpopulation (Figure 4I), which showed that, after PCV2d infection, fewer subpopulations of cells differentiated into the *CD4HelperT*, resulting in a smaller number of cells involved in the immune response *in vivo*, which may be related to the immune escape of PCV2d. Similarly, we analyzed the proposed temporal analysis of *CD8NaiveT* (Figure 4J), which is capable of directly killing infected cells, and *NK cells*, which are an important component of the innate immune system, are capable of directly killing infected cells through the release of cytotoxic molecules, and can enhance the overall antiviral capacity through interactions with other immune cells (Xu et al., 2023). We did KEGG enrichment for 17 genes in the declining *NK* subpopulation (Figure 4K) and boxplot expression extraction for three genes in the neutrophil activation pathway with the most pronounced differences, Fcer1g, Tyrobp and Ccl5 (Figure 4L).

### PCV2d infection leads to abnormalities in activating and interacting with T cells in B cells

**Figure 5:**
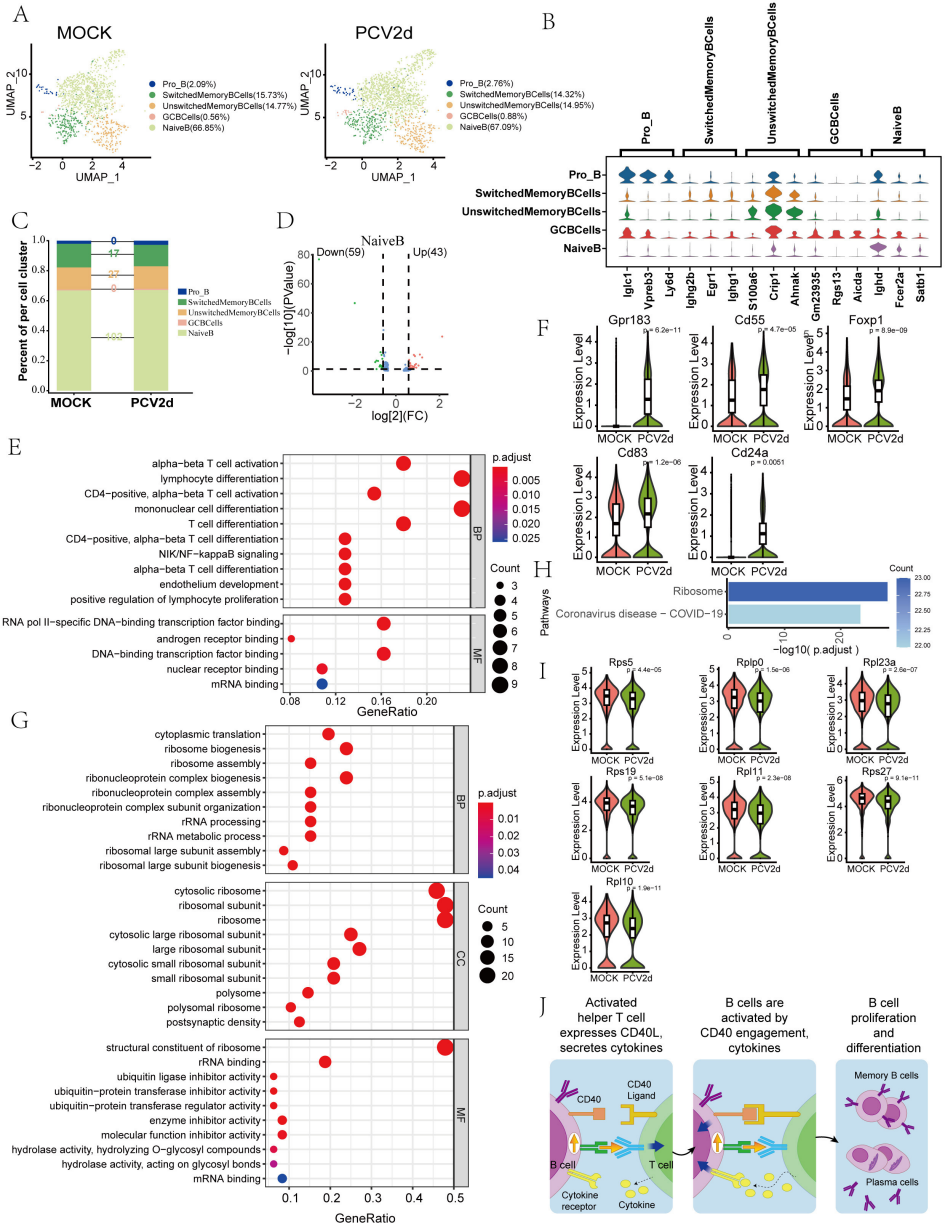
Differential changes in *B cell* occurrence under the two treatment groups. **(A)** Reduced-dimensional grouping of MOCK and PCV2d group *B cell*. **(B)** Five subpopulation subgroups specific to the first three marker genes. **(C)** Occupancy stacking plots and differential gene counts for five subpopulations. **(D)** Volcano maps of up- and down-regulated genes in the *NaiveB* group with large differences. **(E)** GO enrichment results for *NaiveB* upregulated genes (KEGG results without upregulated gene enrichment). **(F)** Expression profiles of genes in the T cell activation pathway. Statistical significance of measurements was assessed using unpaired two-tailed *T*-tests. **(G)** GO enrichment results for *NaiveB* down-regulated genes. **(H)** KEGG enrichment results for *NaiveB* down-regulated genes. **(I)** Down-regulation of ribonucleoprotein assembly gene boxplot expression in the enrichment pathway. Statistical significance of measurements was assessed using unpaired two-tailed *T*-tests. **(J)** B cell and T cell interactions after infection by PCV2d.

The role of B cells in PCV2d infection is an important area of current research, and the critical role of B cells in antibody production and adaptive immune response has brought much attention to their role in PCV2d infection. Secondary fractionation of mouse B cells and subdivision annotation yielded five different subtypes (Figure 5A), including *Pro-B cells* (*Pro_B*, 2.09% in the MOCK group and 2.76% in the PCV2d group), *Switched memory B cells* (*SwitchedMemoryBCells*, 15.73% in the MOCK group and 14.32% in the PCV2d group), *Unswitched memory B cells* (*UnswitchedMemoryBCells*, 14.77% in the MOCK group and 14.95% in the PCV2d group), *Germinal center B cells* (*GCBCells*, 0.56% in the MOCK group and 0.88% in the PCV2d group), *Naive B cells* (*NaiveB*, 66.85% in the MOCK group and 67.09% in the PCV2d group). The specific marker genes for each subpopulation are shown (Figure 5B), the top three specific marker genes for *Pro_B* are Iglc1, Vpreb3 and Ly6d, for *SwitchedMemoryBCells* are Ighg2b, Egr1 and Ighg1, for *UnswitchedMemoryBCells* are S100a6, Crip1 and Ahnak, for *GCBCells* are Gm23935, Rgs13 and Aicda, for *NaiveB* are Ighd, Fcer2a and Satb1. The number of differential genes counted for the five subpopulations infected by PCV2d is shown (Figure 5C). For further analysis of the *NaiveB* subpopulation with the most pronounced differences, we counted the differential gene volcano plots of this subpopulation (Figure 5D), which showed that there were 59 genes decreased and 43 genes up-regulated after PCV2d infection. Firstly, we did GO enrichment for up-regulated genes (Figure 5E), and for up-regulated genes we selected the T cell activation pathway, and did boxplot expression extraction for five genes on the pathway, Gpr183, Cd55, Foxp1, Cd83 and Cd24a (Figure 5F), which were all up-regulated to different degrees after PCV2d infection. B cells combat PCV2d infection by secreting antibodies that neutralize the virus and prevent it from entering target cells (Kiss et al., 2021). We also did GO enrichment (Figure 5G) and KEGG enrichment (Figure 5H) for the decreased genes of the subpopulation, which were enriched in the pathways of cytoplasmic translation, ribosome assembly, etc. We did boxplot expression of seven genes on the ribosome assembly pathway, Rps5, Rplp0, Rpl23a, Rps19, Rpl11, Rps27 and Rpl10 extraction (Figure 5I), all of which were down-regulated to different degrees after PCV2d infection, and we hypothesized that there was a certain down-regulation of the level of antibody production after PCV2d infection, so it led to the immune escape of PCV2d virus. B cells also play an essential role in the regulation of the immune system, and they regulate the whole immune response by secreting cytokines and interacting with T cells directly intensity and nature of the immune response (Rakibuzzaman et al., 2020). We further analyzed the interactions between B cells and T cells (Figure 5J). B cells bind by secreting Cd40 and Cd40 ligands on T cells, and after binding, B cells are activated, complete proliferation and differentiation, and differentiate into memory B cells and plasma cells to participate in the immune response. CD40-CD40L is an important molecular pair in immune regulation, mainly involved in the interaction between T cells and antigen-presenting cells. In PCV2d infection, CD40-CD40L may act through the following mechanism: the CD40-CD40L signaling pathway can activate B cells and T cells, promote the production of antiviral antibodies and cellular immune responses. PCV2d infection may lead to upregulation of CD40L expression, thereby enhancing the host's antiviral immunity (Bereta et al., 2004); The CD40-CD40L signaling pathway plays an important role in inflammatory response and PCV2d infection may induce excessive inflammatory response and tissue damage by regulating this pathway (Borghetti et al., 2013); PCV2d may weaken the host's immune response and promote sustained viral infection by inhibiting the CD40-CD40L signaling pathway (Chen et al., 2023).

### PCV2d infection leads to abnormalities in IL-17 signaling pathway in neutrophils

**Figure 6:**
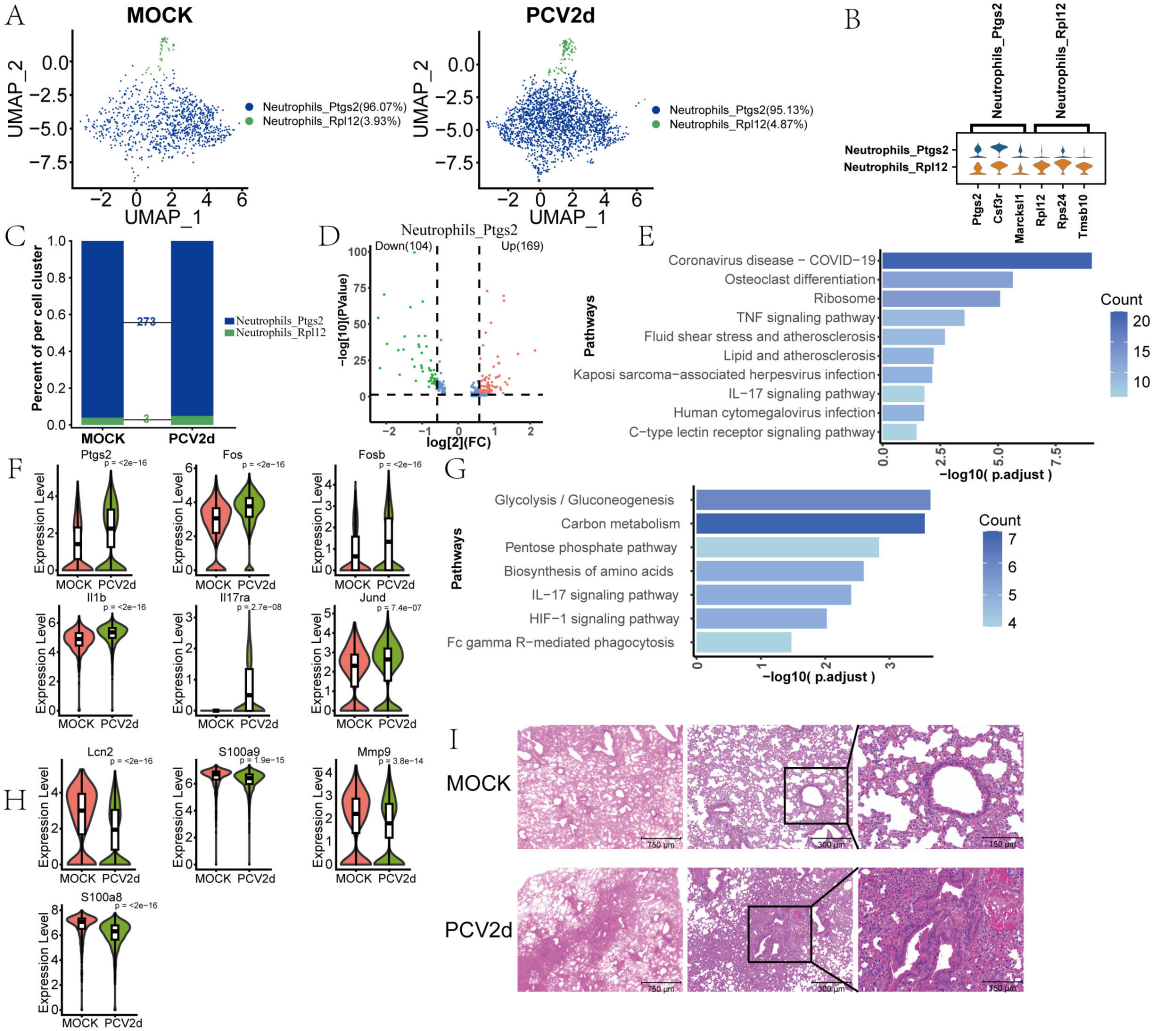
Differential changes in *Neutrophils* under the two treatment groups. **(A)** Downscaling grouping of MOCK and PCV2d group *Neutrophils*. **(B)** Specific top three marker genes for two subpopulation subgroups. **(C)** Occupancy stacking plots and differential gene counts for the two subpopulations. **(D)** Volcano maps of up- and down-regulated genes in the highly differentiated *Neutrophils_ptgs2* group. **(E)** Results of KEGG enrichment of up-regulated genes. **(F)** Boxplot expression of up-regulated genes in the IL-17 pathway. Statistical significance of measurements was assessed using unpaired two-tailed *T*-tests. **(G)** Results of KEGG enrichment of down-regulated genes. **(H)** IL-17 pathway boxplot expression of down-regulated genes. Statistical significance of measurements was assessed using unpaired two-tailed *T*-tests. **(I)** The results of lung sections from both treatment groups showed significant inflammatory cell infiltration in the PCV2d-infected group.

The role of neutrophils in PCV2d infection, especially in lung tissues, has gained significant attention. Neutrophils are key cells of the innate immune response and play an important role in viral infection. Transcriptome sequencing results showed that in mice not infected with PCV2d virus, neutrophils (Neutrophils) were mainly divided into two subpopulations: *Neutrophils_Ptgs2* (96.07%) and *Neutrophils_Rpl12* (3.93%). And after infection with PCV2d virus, the percentage of these two subgroups changed to *Neutrophils_Ptgs2* (95.13%) and *Neutrophils_Rpl12* (4.87%), respectively (Figure 6A). We show that two subpopulations of specific marker genes (Figure 6B), the top three specific marker genes for *Neutrophils_Ptgs2* are Ptgs2, Csf3r and Marcksl1, for *Neutrophils_Rpl12* are Rpl12, Rps24 and Tmsb10. More analysis revealed that the most obvious differences in the two subgroups appeared in the *Neutrophils_Ptgs2* subgroup, with a total of 273 differential genes (Figure 6C), the number of differential genes in subpopulation *Neutrophils_Rpl12* was only three (Retnlg, S100a11 and Lcn2), so the differences in this subpopulation will not be discussed further here. Volcano plot analysis of the differential genes was carried out (Figure 6D), of which 169 were up-regulated genes and 104 were down-regulated genes. The up-regulated differential genes were analyzed by KEGG enrichment (Figure 6E), and we also did the GO enrichment (Supplementary Figure S2J). The results showed that the IL-17 signaling pathway was involved. Boxplot expression was extracted for Ptgs2, Fos, Fosb, Il17ra, Il1b and Jund genes in the pathway (Figure 6F). The down-regulated differential genes were analyzed by KEGG enrichment (Figure 6G), and GO enrichment is done (Supplementary Figure S2K), which is still enriched to the IL-17 signaling pathway, and boxplot expression was extracted for Mmp9, S100a9, Mapk13, S100a8 and Lcn2 genes in the pathway (Figure 6H). In addition, the results of pathological sections showed (Figure 6I) that PCV2d-infected group of mice showed significant inflammatory cell infiltration *in vivo*, with neutrophils migrating to the lungs, which enhanced the immune response, but this may also lead to the expansion of inflammation and cause further damage to the lung tissues (Klesney-Tait et al., 2013). This further confirms that PCV2d virus infection causes an inflammatory response in mice.

### PCV2d infection leads to abnormalities in antigen processing and presentation pathway in endothelial cells

**Figure 7:**
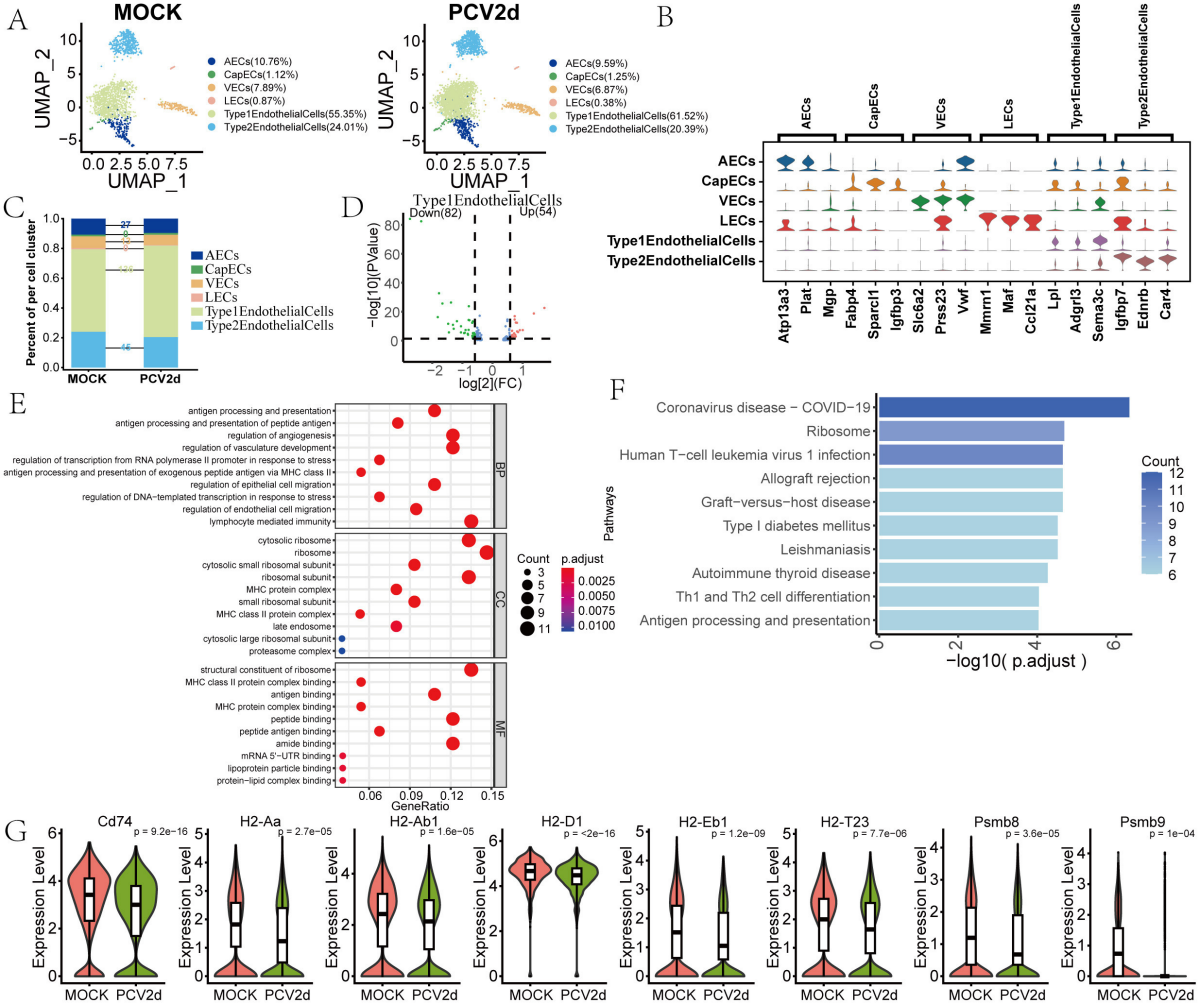
Differential changes in *Endothelial cells* under the two treatment groups. **(A)** Reduced-dimensional grouping of MOCK and PCV2d group *ECs*. **(B)** Six subpopulation subgroups specific to the first three marker genes. **(C)** Occupancy stacking diagrams and differential gene counts for the six subpopulations. **(D)** Volcano maps of up- and down-regulated genes in the *Type 1 Endothelialcell* group with large variance. **(E)** GO enrichment results for genes down-regulated in the *Type 1 Endothelialcell* subpopulation. **(F)** KEGG enrichment results for genes down-regulated in the *Type 1 Endothelialcell* subpopulation. **(G)** Boxplot expression maps of genes in antigen presentation and processing pathways involved in endothelial cells. Statistical significance of measurements was assessed using unpaired two-tailed *T*-tests.

In PCV2d-infected lung tissues, the role of endothelial cells in the pathologic process is gaining attention. Studies have shown that endothelial cells may play an important role in the disease process through mechanisms such as modulation of vascular permeability, participation in inflammatory response and migration of immune cells. Endothelial cells from healthy and infected mice were secondarily fractionated and annotated to obtain six different subtypes (Figure 7A), including *Arterial endothelial cells* (*AECs*, 10.76% in the MOCK group and 9.59% in the PCV2d group), *Capillary endothelial cells* (*CapECs*, 1.12% in the MOCK group and 1.25% in the PCV2d group), *Venous endothelial cells* (*VECs*, 7.89% in the MOCK group and 6.87% in the PCV2d group), *Lymphatic endothelialcells* (*LECs*, 0.87% in the MOCK group and 0.38% in the PCV2d group), *AlveolarCapillaryType1EndothelialCells* (55.35% in the MOCK group and 61.52% in the PCV2d group), *AlveolarCapillaryType2EndothelialCells* (24.01% in the MOCK group and 20.39% in the PCV2d group). The six different subgroups of the specific marker genes are shown (Figure 7B), the top three specific marker genes for *AECs* are Atp13a3, Plat and Mgp, for *CapECs* are Fabp4, Sparcl1 and Igfbp3, for *VECs* are Slc6a2, Prss23 and Vwf, for LECs are Mmrn1, Maf and Ccl21a, for *AlveolarCapillaryType1EndothelialCells* are Lpl, Adgrl3 and Sema3c, for *AlveolarCapillaryType2EndothelialCells* are Igfbp7, Ednrb and Car4. The number of differential genes of the six subpopulations of cells infected by PCV2d was counted (Figure 7C), and the results showed that the number of differential genes was higher in *Type1EndothelialCells*. The gene differential volcano plot of *Type1EndothelialCells* is shown (Figure 7D), and the down-regulated genes accounted for a larger proportion. Therefore, statistically the down-regulated genes in *Type1EndothelialCells* were enriched in GO (Figure 7E) and KEGG (Figure 7F), and the up-regulated genes GO and KEGG were enriched (Supplementary Figures S3A, B). Enrichment to the immunity-related antigen presentation and processing pathway, to the pathway H2-T23, Psmb9, H2-D1, H2-Aa, Psmb8, H2-Ab1, H2-Eb1 and Cd74 genes were extracted for expression (Figure 7G), and the genes in the infected group were down-regulated and expressed to varying degrees. Lung inflammation triggered by PCV2d infection leads to endothelial cell dysfunction, which in turn promotes lung tissue damage and pathology. In addition, viral infection may directly or indirectly lead to endothelial cell damage or apoptosis, thereby exacerbating the inflammatory response and tissue damage in the lungs (Niethamer et al., 2020). We further did a proposed time-series analysis of endothelial cells, the distribution of healthy and infected groups in the proposed temporal trajectories is shown (Supplementary Figure S3C), and the PAGA proposed temporal analysis network diagram and corresponding UMAP are shown (Supplementary Figure S3D), with lines (solid, dashed) indicating the strength of the differentiation relationship.

### PCV2d infection leads to abnormalities in the humoral immune response pathway in epithelial cells

**Figure 8:**
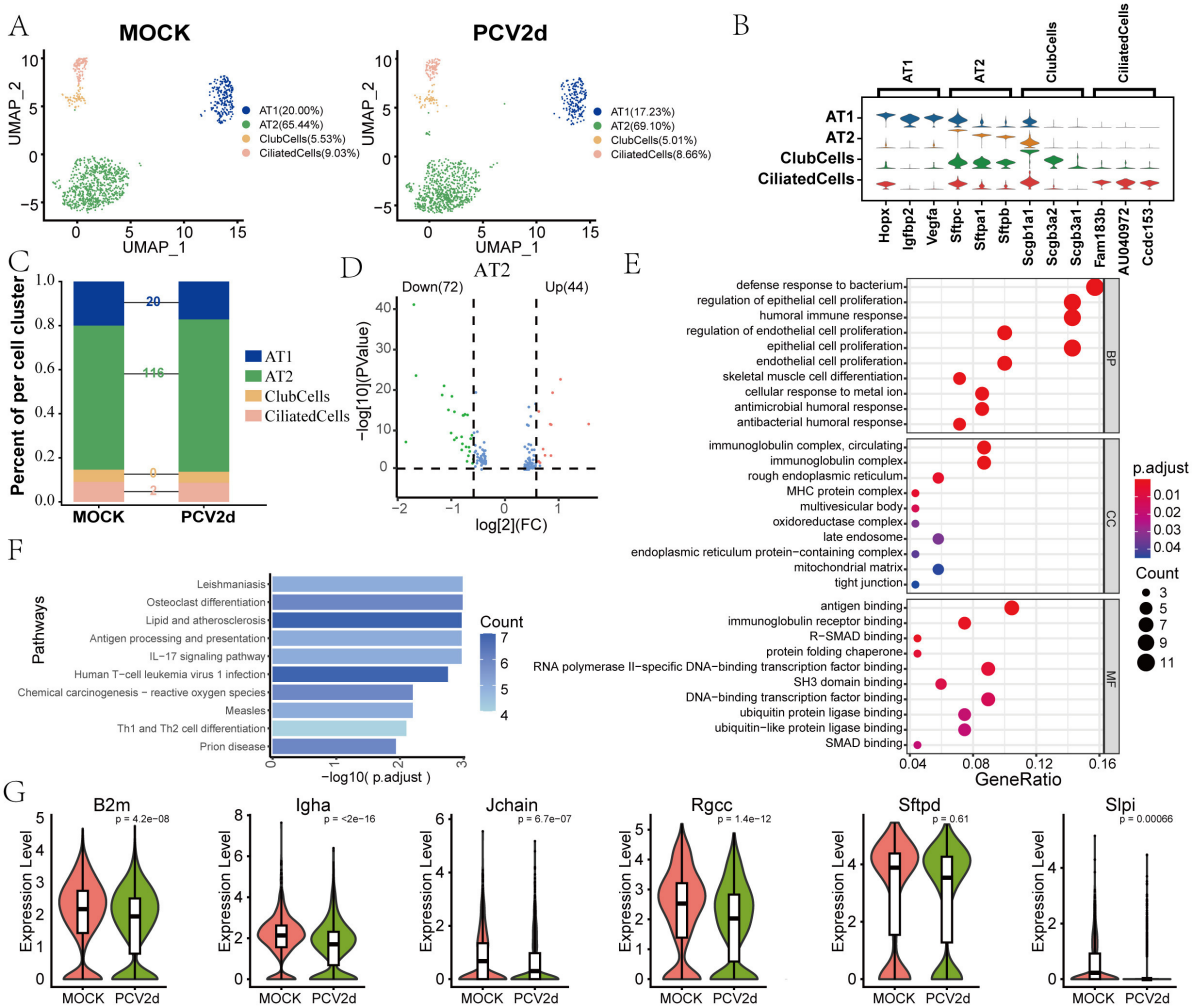
Differential changes in *Epithelialcell* occurrence under the two treatment groups. **(A)** Downscaling and subgrouping of *Epithelialcells* in MOCK and PCV2d groups. **(B)** Specific top three marker genes for four subpopulation subgroups. **(C)** Occupancy stacking plots and differential gene counts for the four subpopulations. **(D)** Volcano maps of up- and down-regulated genes in the highly differentiated *AT2* group. **(E)** GO enrichment results for *AT2* down-regulated genes. **(F)** KEGG enrichment results for *AT2* down-regulated genes. **(G)** Boxplot expression of genes involved in the humoral immune response pathway in epithelial cells. Statistical significance of measurements was assessed using unpaired two-tailed *T*-tests.

Epithelial cells serve as the first line of defense in the respiratory tract and are primary targets for initial viral infection, highlighting the importance of investigating their role in PCV2d pathogenesis. Secondary fractionation of mouse epithelial cells and subdivision annotation yielded four different subtypes (Figure 8A), including *Alveolar type I cells* (*AT1*, 20.00% in the MOCK group and 17.23% in the PCV2d group), *Alveolar type II cells* (*AT2*, 65.44% in the MOCK group and 69.10% in the PCV2d group), *Club cells* (*ClubCells*, 5.53% in the MOCK group and 5.01% in the PCV2d group), *Ciliated cells* (*CiliatedCells*, 9.03% in the MOCK group and 8.66% in the PCV2d group). The specific marker genes for the four subpopulations of cells downgraded into clusters are shown (Figure 8B), the top three specific marker genes for *AT1* are Hopx, Igfbp2 and Vegfa, for *AT2* are Sftpc, Sftpa1 and Sftpb, for *ClubCells* are Scgb1a1, Scgb3a2 and Scgb3a1, for *CiliatedCells* are Fam183b, AU040972 and Ccdc153. We further counted the number of differential genes in the four subpopulations after infection by PCV2d (Figure 8C), and the results showed that the number of *AT2* differential genes was the highest. Therefore, we counted the differential gene volcano plots of this subpopulation of cells (Figure 8D), in which the down-regulated genes accounted for a larger proportion of the genes, and therefore counted the GO (Figure 8E) and KEGG (Figure 8F) enrichment of the down-regulated genes, and GO enrichment and KEGG enrichment of the up-regulated genes, as shown (Supplementary Figures S3E, F), in *AT2*, which were enriched to the humoral immune response pathway. After recognizing PCV2d infection, epithelial cells attract immune cells to the site of infection and initiate an innate immune response, which is an important part of defense against the virus, but an excessive immune response may also lead to tissue damage (Domm et al., 2017). Boxplot expression extraction (Figure 8G) of B2m, Igha, Jchain, Rgcc, Sftpd and Slpi genes in the pathway showed varying degrees of down-regulated expression of genes in the infected group. The proposed chronological analysis (Supplementary Figure S3G) and PAGA temporal trajectory analysis network diagrams of the four subpopulations in the epithelium and the corresponding UMAP are shown (Supplementary Figure S3H), and the lines (solid, dashed) indicate the strength of the differentiation relationship. This suggests an aberrant process of differentiation of the *AT2* subpopulation to the other three subpopulations following PCV2d infection.

### PCV2d infection leads to abnormalities in IL-17 signaling pathway in fibroblasts

**Figure 9:**
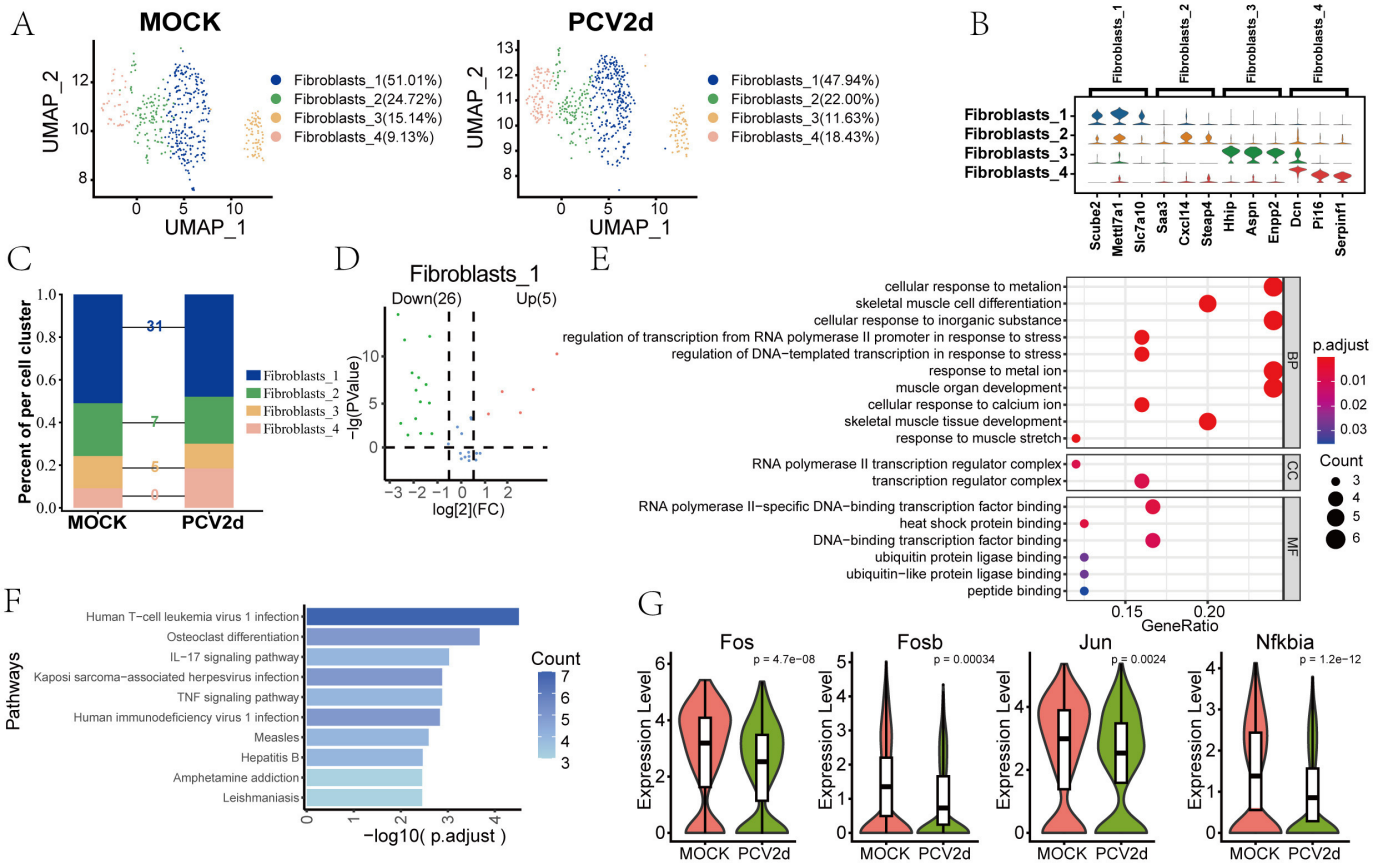
Differential changes in the occurrence of *Fibroblasts* under the two treatment groups. **(A)** Downscaling and grouping of *Fibroblasts* in MOCK and PCV2d groups. **(B)** Specific top three marker genes for four subpopulation subgroups. **(C)** Occupancy stacking plots and differential gene counts for the four subpopulations. **(D)** Volcano maps of up- and down-regulated genes in the highly differentiated *Fibroblasts_1* group. **(E)** GO enrichment results of *Fibroblasts_1* down-regulated genes. **(F)** KEGG enrichment results of Fibroblasts_1 down-regulated genes. **(G)** Fibroblasts downregulate gene boxplot expression in the gene-enriched IL17 pathway. Statistical significance of measurements was assessed using unpaired two-tailed *T*-tests.

The role of fibroblasts in PCV2d infection has not been extensively studied, and we elaborated on the regulation of inflammatory responses by fibroblasts at the single-cell level. Secondary binning of mouse fibroblasts yielded four subpopulations (Figure 9A), *Fibroblasts_1* (51.01% in the MOCK group and 47.94% in the PCV2d group), *Fibroblasts_2* (24.72% in the MOCK group and 22.00% in the PCV2d group), *Fibroblasts_3* (15.14% in the MOCK group and 11.63% in the PCV2d group), and *Fibroblasts_4* (9.13% in the MOCK group and 18.43% in the PCV2d group). Statistics of the specific marker genes for the four subpopulations of the descending subpopulations are shown (Figure 9B), the top three specific marker genes for *Fibroblasts_1* are Scube2, Mettl7a1 and Slc7a10, for *Fibroblasts_2* are Saa3, Cxcl14 and Steap4, for *Fibroblasts_3* are Hhip, Aspn and Enpp2, for *Fibroblasts_4* are Dcn, Pi16 and Serpinf1. Further statistics of the four subpopulations were analyzed for differential genes after being infected by PCV2d (Figure 9C), and the results showed that *Fibroblasts_1* had the most differential genes. Therefore, the volcano plot of differential genes of the statistical subpopulations (Figure 9D), the down-regulated genes accounted for more significant proportion. Therefore, the GO (Figure 9E) and KEGG (Figure 9F) enrichment of statistically down-regulated genes, fibroblasts can be involved in local inflammatory responses by secreting a variety of cytokines, and PCV2d infection may activate fibroblasts, which may amplify or modulate inflammatory responses in lung tissue. We focused on the IL-17 signaling pathway and did a boxplot expression extraction of Fos, Fosb, Jun and Nfkbia genes in the pathway, and the results are shown (Figure 9G), and all of them turned out to be downregulated to a certain extent. In addition, the proposed chronological analysis (Supplementary Figure S3I) and PAGA proposed chronological analysis network diagrams and corresponding UMAPs under two treatment groups for the four subpopulations are shown (Supplementary Figure S3J), and the lines (solid, dashed) indicate the strength of the differentiation relationship. We hypothesize that under certain circumstances, fibroblasts may provide a “hideout” for the virus to evade host immune surveillance, thereby promoting persistent infection of the virus to achieve the immune escape mechanism of PCV2d.

### Cell-to-cell communication under two treatment groups

**Figure 10:**
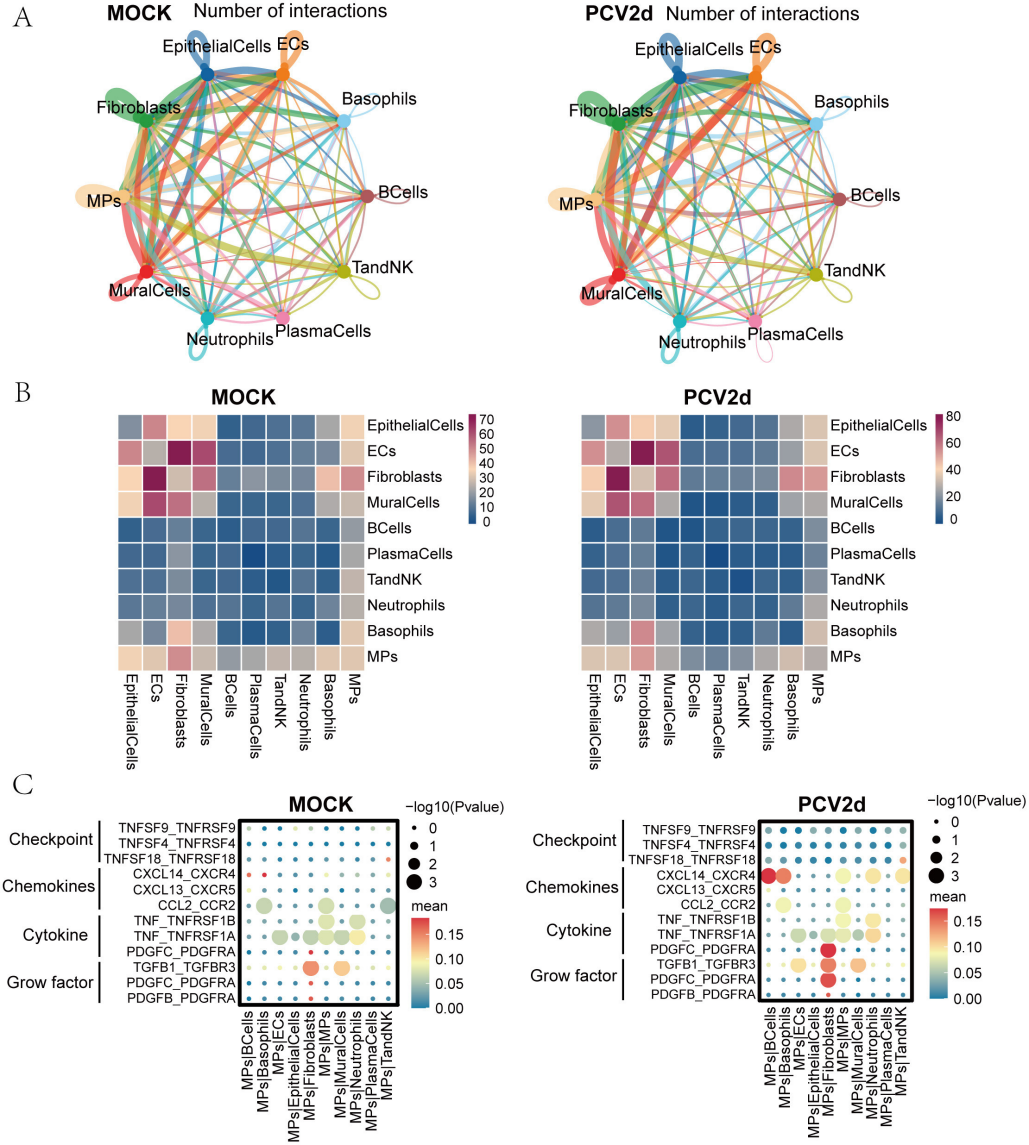
Communication of subpopulations of cells under the two treatment groups. **(A)** Demonstration of a network of interactions between 10 subpopulations under healthy control and PCV2d-infected groups. **(B)** Heatmap of cell type interactions under healthy control and PCV2d-infected groups. **(C)** Interaction of monocyte macrophage subsets as donors with chemokines, immune checkpoints, cytokines and growth factors, including self and nine other subsets, under healthy control and PCV2d-infected groups.

Intercellular communication affects a variety of biological processes in multicellular organisms, such as development, tissue homeostasis (Valls and Esposito, 2022), and immune responses (Altan-Bonnet and Mukherjee, 2019). Dysregulated intercellular communication and cancer (Graeber and Eisenberg, 2001; Hu et al., 2021) is related to diseases like aging. Single-cell RNA-seq-based intercellular communication analysis was combined with spatial transcriptomics and multi-omics to analyze the differences in intercellular communication after PCV2d infection of mouse lung tissues through more comprehensive datasets. Firstly, for the interactions network map between all cell types under both treatment groups (Figure 10A), the line colors were consistent with the ligand cell types, and the line thickness was positively correlated with the number of interactions. We further did statistics on the cell type interactions heatmap (Figure 10B), showing the number of interaction pairs between each two cell types, the darker the color, the higher the number of interactions pairs between two cell types.

Immune checkpoints, chemokines, cytokines, and growth factors act as costimulatory molecules for T-cell activation, and macrophages can produce a variety of cytokines that counterbalance immune-mediated tissue damage with repair and homeostasis maintenance (Kiss et al., 2021). Under the PCV2d takedown treatment group, there were more pronounced differences in the immune checkpoint pathways compared to the control group, mainly in the CTLA4_CD80 and CD28_CD80 pathways that interact with monocyte macrophages (Figure 10C). In the signaling pathway analysis of monocyte macrophages and other subpopulations, the more obvious differences were in chemokines, cytokines, and growth factors, specifically in the extensive CXCL14_CXCR4 pathway differences, which have previously been shown to be associated with fibroblast generation and activation of the CXCL14_CXCR4 chemokine axis in IPF lungs (Figure 10C; Rodriguez et al., 2018), CXCL14 is a chemokine that mainly participates in the recruitment of immune cells and the regulation of inflammatory responses by binding to its receptor CXCR4. In viral infection, the CXCL14-CXCR4 axis may affect PCV2d infection through the following mechanisms. CXCL14 can recruit immune cells to the site of infection and enhance antiviral immune response. Research has shown that PCV2d infection can induce local inflammatory responses, and CXCL14 may play an important role in this process (Sidahmed et al., 2014); CXCR4 can be utilized by certain viral infections, promoting virus entry into cells or enhancing virus replication (Fernandis et al., 2003); PCV2d may inhibit the host's antiviral immune response and facilitate immune evasion by regulating the CXCL14-CXCR4 signaling pathway (Franzo et al., 2016). The more obvious cytokine and growth factor pathway difference is manifested as the PDGFC_PDGFRA pathway. Monocyte macrophages recognize and phagocytose pathogens during infection and modulate the local immune response by secreting cytokines (e.g., TNF, PDGFC). These cytokines can help mobilize other immune cells to participate in the response, but may also lead to overactivation of the inflammatory response (Meng, 2013; Fehér et al., 2023). Communication between the other nine cells is shown (Supplementary Figures S4A–I).

## Discussion

PCV2 intensifies the spreading of severe porcine syndromes worldwide, causing immunosuppression and co-morbidity with other dangerous pathogens, leading to severe economic loss in the swine industry (Mo et al., 2019).

**Figure 11:**
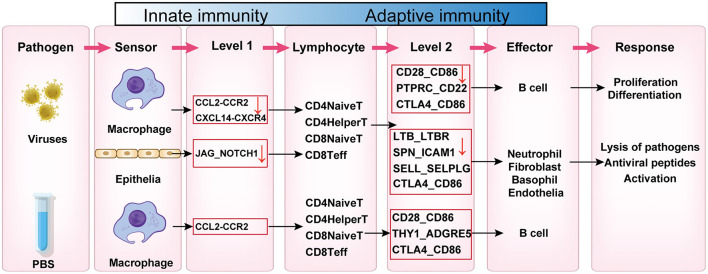
The mechanism of innate immunity to adaptive immunity in the body after infection with PCV2d virus.

In this study, we performed a detailed analysis of various subpopulations in lung tissues of PCV2d-infected mice using single-cell sequencing. The results showed that PCV2d infection induced significant changes in multiple cellular subpopulations, revealing the complex effects of viral infection on lung tissues (Figure 11). We observed significant remodeling of immune cell subpopulations, particularly significant changes in the proportions of neutrophils, macrophages, and T cells. Macrophages exhibited a stronger inflammatory response after infection, which is consistent with previous studies suggesting that macrophages play a key role in viral clearance and inflammatory regulation. Most notably, they affected antigen presentation and processing, and multiple subpopulations of cells *in vivo* were involved in this process, which may be analyzed in relation to the immune escape mechanism of PCV2d virus. At the same time, the subpopulation distribution of T cells was also altered, suggesting that PCV2d infection may have affected the differentiation and activation status of T cells. Second, the cellular status in lung epithelial cells was also altered by PCV2d infection. Infection with PCV2d first activates innate immunity in the organism, which in turn activates an adaptive immune response through intercellular interactions. Specifically, after PCV2d infection of tissues, macrophages and epithelial cells first participate in the immune response. macrophages activate several subpopulations of T cells through the CCL2-CCR2 axis, and epithelial cells activate T cells through the JAG_NOTCH1 axis. After being activated, the T cells generate a secondary response, which activates T cells through the CD28-CD86, PTPRC_CD22, respectively. CTLA4_CD86 interacts with B cells and thus activates the immune response of B cells, and through LTB_LTBR, SPN_ICAM1, SELL_SELPLG, CTLA4_CD86 interacts with neutrophils, fibroblasts, and basophils and activates the response of the target cells, and ultimately, adaptive immunity manifests itself in the form of cell proliferation, differentiation, and the response to the pathogen lysis and production of antiviral peptides. We found that infection resulted in a partial population of epithelial cells exhibiting upregulation of stress response markers, which may be related to direct viral damage to host cells. In addition, changes in stromal cell subpopulations further supported structural and functional alterations in lung tissue that may be associated with the fibrotic process triggered by infection.

Overall, this study revealed the multilevel effects of PCV2d infection on mouse lung tissue by single-cell sequencing. Future studies can further explore the specific mechanisms of these cellular subpopulation changes and their role in PCV2d-associated pathologic processes. This will provide an important reference for understanding the pathological mechanisms of PCV2d infection and developing potential therapeutic strategies.

## Materials and methods

### Isolation and identification of PCV2

In 2023, lymph samples of piglets with respiratory diseases and emaciation were collected from a large-scale pig farm in Shandong. Take 1 g from the sample, add 1 × PBS containing 200 IU/mL penicillin and 0.2 mg/mL streptomycin respectively, freeze and thaw 3 times after grinding treatment, centrifuge at 10 000 rpm for 30 min, and the supernatant was filtered and decontaminated by 0.22 μm pore size filter, and then 500 μL of each of the filtered and decontaminated grinding solution was inoculated into PK-15 cells cultured in T25 culture flasks, with cell confluence of 70–80%, adsorbed in 37°C, 5% CO_2_ cell culture box for 2 h, shaken every 30 min, and discarded after adsorption. The cells were inoculated into PK-15 cells cultured in T25 culture flasks with 70–80% cell confluence, and then adsorbed for 2 h at 37°C in a 5% CO_2_ cell culture incubator with shaking every 30 min. After adsorption, the cells were discarded, and 5 mL of DMEM (Dulbecco's modified Eagle medium, Gibco, 2393822) medium containing 2% FBS (heat-inactivated fetal bovine serum, LONSERA, S711-001) was added to continue the culture. Seventy-two hours later, the cells were frozen and thawed three times with supernatant, and then centrifuged and the supernatant harvested for the F1 generation. 500 μL of the F1 generation was inoculated into PK-15 cells for 5 generations, and the cells were blindly passaged for 5 generations. Cells were inoculated with PK-15 cells for 5 generations, and the cultures were stored at −80°C. The sequence of the PCV2d strain was submitted to NCBI, with the GenBank accession number OQ730503.

### Pathogenicity studies in mice

To study the pathogenicity of the isolates in mammals, the 6th-week-old healthy BALB/c mice were selected for infection test. Mice were purchased from the Chengdu Dossy Experimental Animals CO. (Chengdu, China) and we promise that the study was performed according to the international, national and institutional rules considering animal experiments, clinical studies and biodiversity rights. The study protocol was approved by the Institutional Animal Care and Use Committee of Northwest A&F University. The mice were randomly divided into 2 groups, and infected with PCV2d (1 × 10^5.14^ TCID_50_/0.1 ml). The control mice were infected with PBS.

### Tissue dissociation and single-cell suspension preparation

The fresh tissues were stored in the sCelLiveTM Tissue Preservation Solution (Singleron) on ice after the surgery within 30 min. The specimens were washed with Hanks Balanced Salt Solution (HBSS) for three times, minced into small pieces, and then digested with 3 mL sCelLiveTM Tissue Dissociation Solution (Singleron) by Singleron PythoN™ Tissue Dissociation System at 37°C for 15 min. The cell suspension was collected and filtered through a 40-micron sterile strainer. Afterwards, the GEXSCOPE^®^ red blood cell lysis buffer (RCLB, Singleron) was added, and the mixture [Cell: RCLB=1:2 (volume ratio)] was incubated at room temperature for 5–8 min to remove red blood cells. The mixture was then centrifuged at 300 × g 4 °C for 5 min to remove supernatant and suspended softly with PBS.

### Single cell RNA sequencing

Single-cell suspensions (2 × 10^5^ cells/mL) with PBS (HyClone) were loaded onto microwell chip using the Singleron Matrix^®^ Single Cell Processing System. Barcoding Beads are subsequently collected from the microwell chip, followed by reverse transcription of the mRNA captured by the Barcoding Beads and to obtain cDNA, and PCR amplification. The amplified cDNA is then fragmented and ligated with sequencing adapters. The scRNA-seq libraries were constructed according to the protocol of the GEXSCOPE^®^ Single Cell RNA Library Kits (Singleron; Dura et al., 2019). Individual libraries were diluted to 4 nM, pooled, and sequenced on Illumina novaseq 6,000 with 150 bp paired end reads.

### Primary analysis of raw read data (snRNA-seq)

Raw reads were processed to generate gene expression profiles using CeleScope v1.5.2 (Singleron Biotechnologies) with default parameters. Briefly, Barcodes and UMIs were extracted from R1 reads and corrected. Adapter sequences and poly A tails were trimmed from R2 reads and the trimmed R2 reads were aligned against the GRCh38 (hg38) {GRCm38 (mm10)} transcriptome using STAR (v2.6.1b). Uniquely mapped reads were then assigned to genes with FeatureCounts (v2.0.1). Successfully Assigned Reads with the same cell barcode, UMI and gene were grouped together to generate the gene expression matrix for further analysis.

### Quality control, dimension-reduction and clustering (seurat)

Seurat v 3.1.2 was used for quality control, dimensionality reduction and clustering. For each sample dataset, we filtered expression matrix by the following criteria: (1) cells with gene count < 200 or with top 2% gene count were excluded; (2) cells with top 2% UMI count were excluded; (3) cells with mitochondrial content >20% were excluded; (4) genes expressed in < 5 cells were excluded. After filtering, cells were retained for the downstream analyses. Gene expression matrix was normalized and scaled using functions NormalizeData and ScaleData. Top 2,000 variable genes were selected by FindVariableFeatures for PCA analysis. Cells were separated into 18 clusters by FindClusters, using the top 20 principal components and resolution parameter at xxx. Cell clusters were visualized using t-Distributed Stochastic Neighbor Embedding (t-SNE) or Uniform Manifold Approximation and Projection (UMAP) with Seurat functions RunTSNE and RunUMAP.

### Differentially expressed genes (DEGs) analysis (seurat)

To identify differentially expressed genes (DEGs), we used the Seurat FindMarkers function based on Wilcoxon rank sum test with default parameters, and selected the genes expressed in more than 10% of the cells in both of the compared groups of cells and with an average log (Fold Change) value >0.25 as DEGs. Adjusted *p*-value was calculated by Bonferroni Correction and the value 0.05 was used as the criterion to evaluate the statistical significance.

### Pathway enrichment analysis

To investigate the potential functions of different subpopulations of cells, Gene Ontology (GO) and Kyoto Encyclopedia of Genes and Genomes (KEGG) analysis were used with the “clusterProfiler” R package v 3.16.1 (Yu et al., 2012). Pathways with p_adj value < 0.05 were considered as significantly enriched. Selected significant pathways were plotted as bar plots. GSEA was performed on different types of genes in different types of clusters. For GSVA pathway enrichment analysis, the average gene expression of each cell type was used as input data (Hänzelmann et al., 2013). Gene Ontology gene sets including molecular function (MF), biological process (BP), and cellular component (CC) categories were used as reference. Protein-protein interactions (PPI) of DEGs in xxx clusters were predicted based on known interactions of genes with relevant GO terms in the StringDB (1.22.0; Szklarczyk et al., 2019).

### Cell-type recognition with cell-ID

Cell-ID is multivariate approach that extracts gene signatures for each individual cell and perform cell identity recognition using hypergeometric tests (HGT). Dimensionality reduction was performed on normalized gene expression matrix through multiple correspondence analysis, where both cells and genes were projected in the same low dimensional space (Cortal et al., 2021). Then a gene ranking was calculated for each cell to obtain most featured gene sets of that cell. HGT were performed on these gene sets against brain reference from SynEcoSys database, which contains all cell-type's featured genes. Identity of each cell was determined as the cell-type has the minimal HGT *p*-value. For cluster annotation, Frequency of each cell-type was calculated in each cluster, and cell-type with highest frequency was chosen as cluster's identity.

### Cell-cell interaction analysis: cellphoneDB

Cell-cell interaction (CCI) in both cell types were predicted based on known ligand–receptor pairs by Cellphone DB (v2.1.0; Efremova et al., 2020) version. Permutation number for calculating the null distribution of average ligand-receptor pair expression in randomized cell identities was set to 1,000. Individual ligand or receptor expression was thresholded by a cutoff based on the average log gene expression distribution for all genes across each cell type. Predicted interaction pairs with *p* < 0.05 and of average log expression > 0.1 were considered as significant and visualized by heatmap_plot and dot_plot in CellphoneDB.

### Pseudotime trajectory analysis: monocle2

Cell differentiation trajectory of monocyte subtypes was reconstructed with the Monocle2 v 2.10.0 (Qiu et al., 2017). For constructing the trajectory, top 2,000 highly variable genes were selected by Seurat (v3.1.2) FindVairableFeatures, and dimension-reduction was performed by DDRTree. The trajectory was visualized by plot_cell_trajectory function in Monocle2.

### Histopathological analysis

Haematoxylin and eosin (HE) staining analysis was performed to observe the pathological changes in mouse tissues. Tissues fixed in 4% paraformaldehyde solution were sent to the Wuhan Servicebio Technology CO. (Wuhan, China) to produce HE-stained sections for pathohistological observation.

## Data Availability

All relevant data can be found within this article and mice lungs single-cell RNA-seq data is obtained from the NCBI GEO database (GSE278049).

## Supplementary material

Supplementary Figure S1 Enrichment results of differential genes and proposed time series analysis of *MPs* subpopulations. **(A)** GO enrichment results for up-regulated differential genes in the *AlveolarMacro* subpopulation under two treatment groups. **(B)** KEGG enrichment results for up-regulated differential genes in the *AlveolarMacro* subpopulation under two treatment groups. **(C)** GO enrichment results for up-regulated differential genes in the *Macrophages* subpopulation under two treatment groups. **(D)** KEGG enrichment results for up-regulated differential genes in the *Macrophages* subpopulation under two treatment groups. **(E)** Boxplot expression of genes specific to antigen presentation and processing pathways in both subpopulations. Statistical significance of measurements was assessed using unpaired two-tailed *T*-tests. **(F)** GO enrichment results for down-regulated differential genes in the *Monocytes* subpopulation under two treatment groups. **(G)** KEGG enrichment results for down-regulated differential genes in the *Monocytes* subpopulation under two treatment groups. **(H)** Proposed time-series analysis of different subpopulations of macrophage cells under the two treatments, each color representing a different kind of subpopulation.

Supplementary Figure S2 Differential gene enrichment results and paga analysis of *MPs* subgroups. **(A)** PAGA analysis of different subpopulations of macrophage cells, each color representing a different kind of subpopulation. **(B)** GO enrichment results for down-regulated differential genes in the *CD4NaiveT* subpopulation under two treatment groups. **(C)** KEGG enrichment results for down-regulated differential genes in the *CD4NaiveT* subpopulation under two treatment groups. **(D)** GO enrichment results for up-regulated differential genes in the *CD4HelperT* subpopulation under two treatment groups. **(E)** GO enrichment results for down-regulated differential genes in the *CD4HelperT* subpopulation under two treatment groups. **(F)** KEGG enrichment results for down-regulated differential genes in the *CD4HelperT* subpopulation under two treatment groups. **(G)** GO enrichment results for up-regulated differential genes in the *CD8NaiveT* subpopulation under two treatment groups. **(H)** GO enrichment results for down-regulated differential genes in the *CD8NaiveT* subpopulation under two treatment groups. **(I)** GO enrichment results for down-regulated differential genes in the *CD8NaiveT* subpopulation under two treatment groups. **(J)** GO enrichment results for up-regulated differential genes in the *Neutrophils_Ptgs2* subpopulation under two treatment groups. **(K)** GO enrichment results for down-regulated differential genes in the *Neutrophils_Ptgs2* subpopulation under two treatment groups.

Supplementary Figure S3 Differential gene enrichment results and proposed temporal sequence analysis and paga analysis. **(A)** GO enrichment results for up-regulated differential genes in the *Type1EndothelialCells* subpopulation under two treatment groups. **(B)** KEGG enrichment results for up-regulated differential genes in the *Type1EndothelialCells* subpopulation under two treatment groups. **(C)** Proposed time-series analysis of different subpopulations of *Endothelial cells* under the two treatments, each color representing a different kind of subpopulation. **(D)** PAGA analysis of different subpopulations of *Endothelial cells*, each color representing a different kind of subpopulation. **(E)** GO enrichment results for up-regulated differential genes in the *AT2* subpopulation under two treatment groups. **(F)** KEGG enrichment results for up-regulated differential genes in the *AT2* subpopulation under two treatment groups. **(G)** Proposed time-series analysis of different subpopulations of *Epithelial cells* under the two treatments, each color representing a different kind of subpopulation. **(H)** PAGA analysis of different subpopulations of *Epithelial cells*, each color representing a different kind of subpopulation. **(I)** Proposed time-series analysis of different subpopulations of *Fibroblas*t cells under the two treatments, each color representing a different kind of subpopulation. **(J)** PAGA analysis of different subpopulations of *Fibroblast* cells, each color representing a different kind of subpopulation.

Supplementary Figure S4 Cellular communication of the remaining nine subpopulations of cells in the MOCK and PCV2d groups, respectively. **(A)** Communication pathways of *Neutrophils* subpopulations with other subpopulations under the two treatment groups, darker colors represent stronger mutualisms. **(B)** Communication pathways of *TandNK* subpopulations with other subpopulations under the two treatment groups, darker colors represent stronger mutualisms. **(C)** Communication pathways of *Endothelial* subpopulations with other subpopulations under the two treatment groups, darker colors represent stronger mutualisms. **(D)** Communication pathways of *BCells* subpopulations with other subpopulations under the two treatment groups, darker colors represent stronger mutualisms. **(E)** Communication pathways of *Epithelial cell* subpopulations with other subpopulations under the two treatment groups, darker colors represent stronger mutualisms. **(F)** Communication pathways of *Fibroblast* subpopulations with other subpopulations under the two treatment groups, darker colors represent stronger mutualisms. **(G)** Communication pathways of *Plasma cell* subpopulations with other subpopulations under the two treatment groups, darker colors represent stronger mutualisms. **(H)** Communication pathways of *Basophils* subpopulations with other subpopulations under the two treatment groups, darker colors represent stronger mutualisms. **(I)** Communication pathways of *Mural cell* subpopulations with other subpopulations under the two treatment groups, darker colors represent stronger mutualisms.
