# Supplementary figures and images for: The single-cell transcriptional landscape of lung cells from PCV2d-infected mice
Source: Front Microbiol. 2025 Mar 24;16:1554961. doi: 10.3389/fmicb.2025.1554961 (PMC11973356; doi:10.3389/fmicb.2025.1554961)

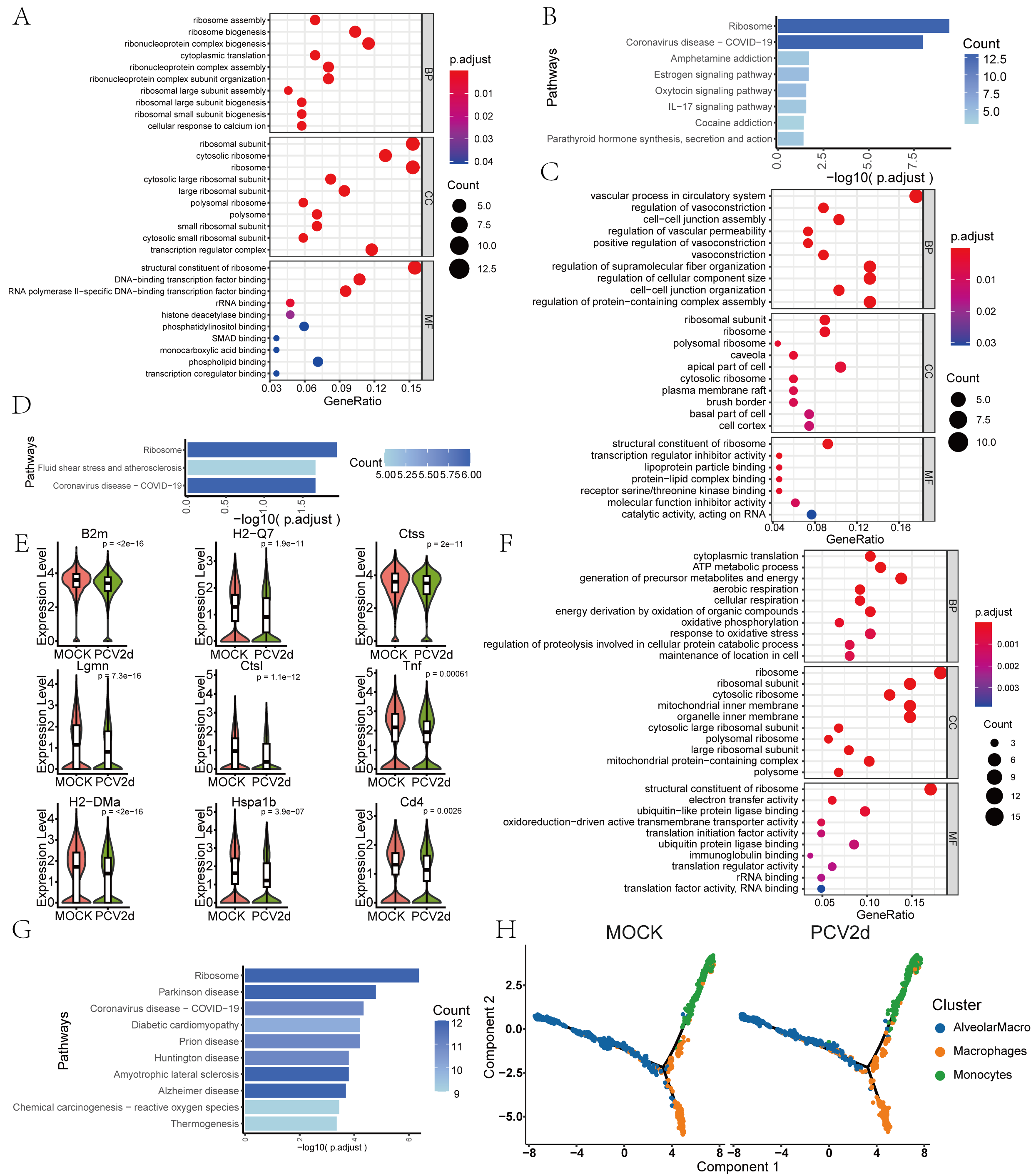

Supplement: Supplementary Figure S1 — Enrichment results of differential genes and proposed time series analysis of MPs subpopulations. (A) GO enrichment results for up-regulated differential genes in the AlveolarMacro subpopulation under two treatment groups. (B) KEGG enrichment results for up-regulated differential genes in the AlveolarMacro subpopulation under two treatment groups. (C) GO enrichment results for up-regulated differential genes in the Macrophages subpopulation under two treatment groups. (D) KEGG enrichment results for up-regulated differential genes in the Macrophages subpopulation under two treatment groups. (E) Boxplot expression of genes specific to antigen presentation and processing pathways in both subpopulations. Statistical significance of measurements was assessed using unpaired two-tailed T-tests. (F) GO enrichment results for down-regulated differential genes in the Monocytes subpopulation under two treatment groups. (G) KEGG enrichment results for down-regulated differential genes in the Monocytes subpopulation under two treatment groups. (H) Proposed time-series analysis of different subpopulations of macrophage cells under the two treatments, each color representing a different kind of subpopulation. [file Image_1.tif]

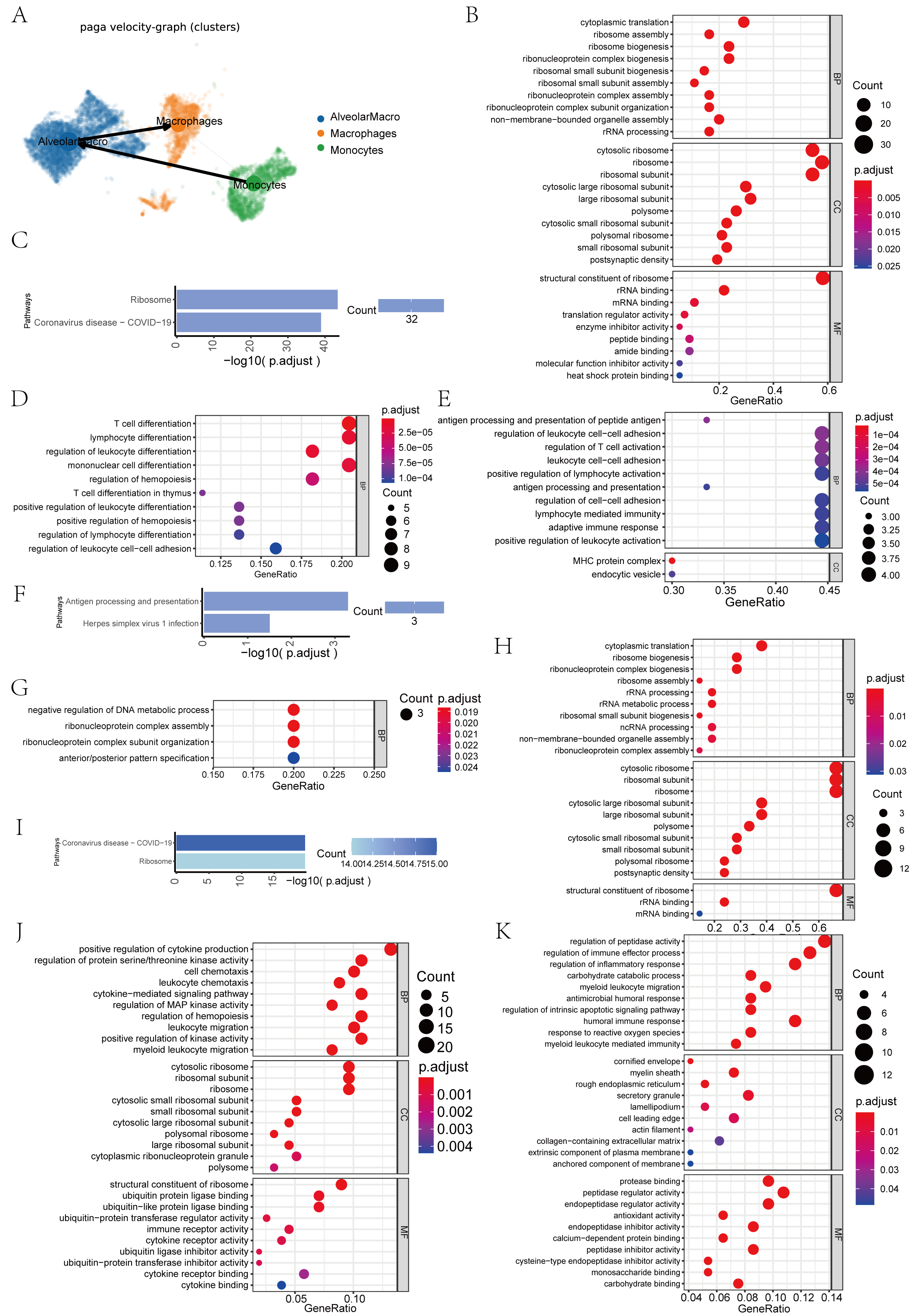

Supplement: Supplementary Figure S2 — Differential gene enrichment results and paga analysis of MPs subgroups. (A) PAGA analysis of different subpopulations of macrophage cells, each color representing a different kind of subpopulation. (B) GO enrichment results for down-regulated differential genes in the CD4NaiveT subpopulation under two treatment groups. (C) KEGG enrichment results for down-regulated differential genes in the CD4NaiveT subpopulation under two treatment groups. (D) GO enrichment results for up-regulated differential genes in the CD4HelperT subpopulation under two treatment groups. (E) GO enrichment results for down-regulated differential genes in the CD4HelperT subpopulation under two treatment groups. (F) KEGG enrichment results for down-regulated differential genes in the CD4HelperT subpopulation under two treatment groups. (G) GO enrichment results for up-regulated differential genes in the CD8NaiveT subpopulation under two treatment groups. (H) GO enrichment results for down-regulated differential genes in the CD8NaiveT subpopulation under two treatment groups. (I) GO enrichment results for down-regulated differential genes in the CD8NaiveT subpopulation under two treatment groups. (J) GO enrichment results for up-regulated differential genes in the Neutrophils_Ptgs2 subpopulation under two treatment groups. (K) GO enrichment results for down-regulated differential genes in the Neutrophils_Ptgs2 subpopulation under two treatment groups. [file Image_2.tif]

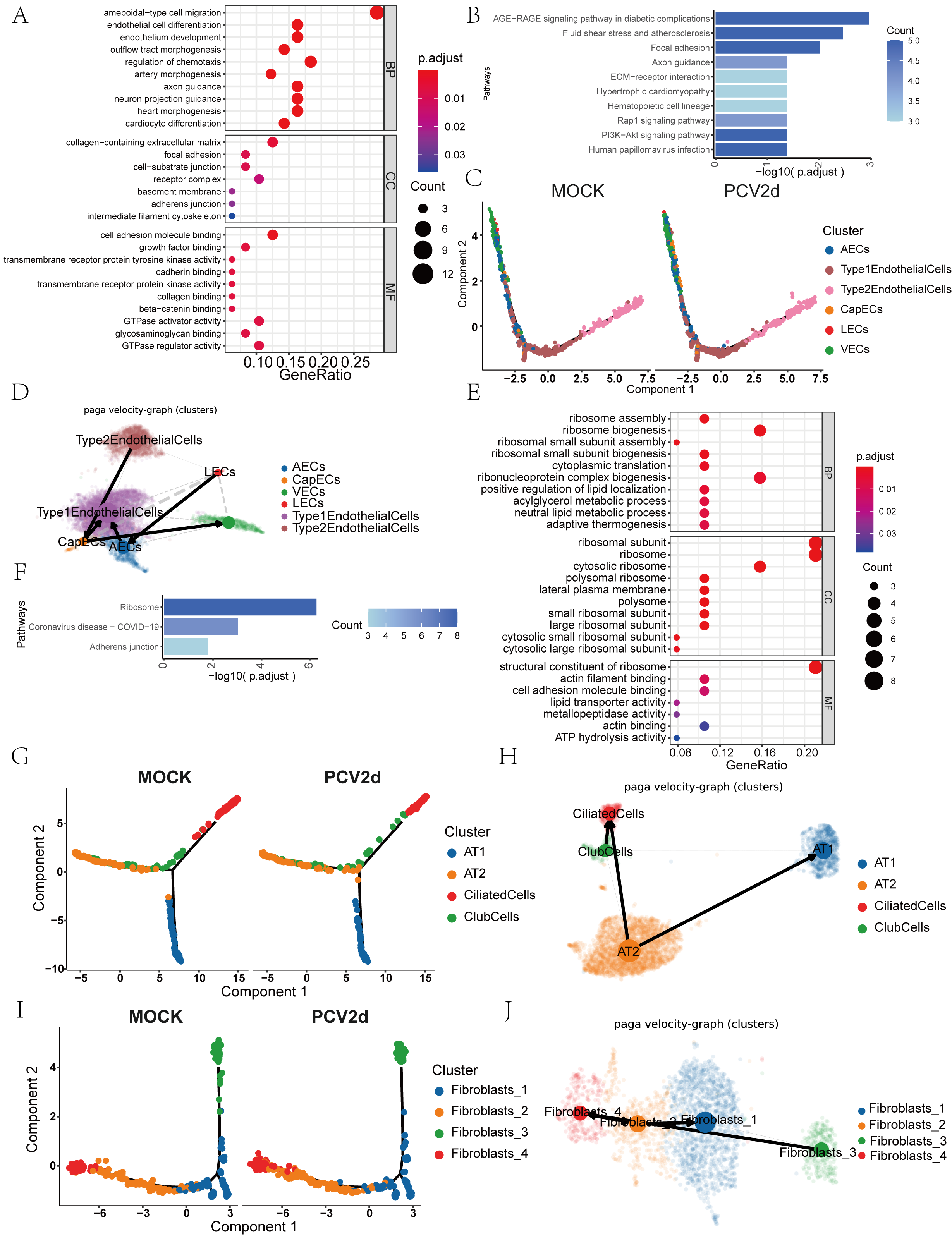

Supplement: Supplementary Figure S3 — Differential gene enrichment results and proposed temporal sequence analysis and paga analysis. (A) GO enrichment results for up-regulated differential genes in the Type1EndothelialCells subpopulation under two treatment groups. (B) KEGG enrichment results for up-regulated differential genes in the Type1EndothelialCells subpopulation under two treatment groups. (C) Proposed time-series analysis of different subpopulations of Endothelial cells under the two treatments, each color representing a different kind of subpopulation. (D) PAGA analysis of different subpopulations of Endothelial cells, each color representing a different kind of subpopulation. (E) GO enrichment results for up-regulated differential genes in the AT2 subpopulation under two treatment groups. (F) KEGG enrichment results for up-regulated differential genes in the AT2 subpopulation under two treatment groups. (G) Proposed time-series analysis of different subpopulations of Epithelial cells under the two treatments, each color representing a different kind of subpopulation. (H) PAGA analysis of different subpopulations of Epithelial cells, each color representing a different kind of subpopulation. (I) Proposed time-series analysis of different subpopulations of Fibroblast cells under the two treatments, each color representing a different kind of subpopulation. (J) PAGA analysis of different subpopulations of Fibroblast cells, each color representing a different kind of subpopulation. [file Image_3.tif]

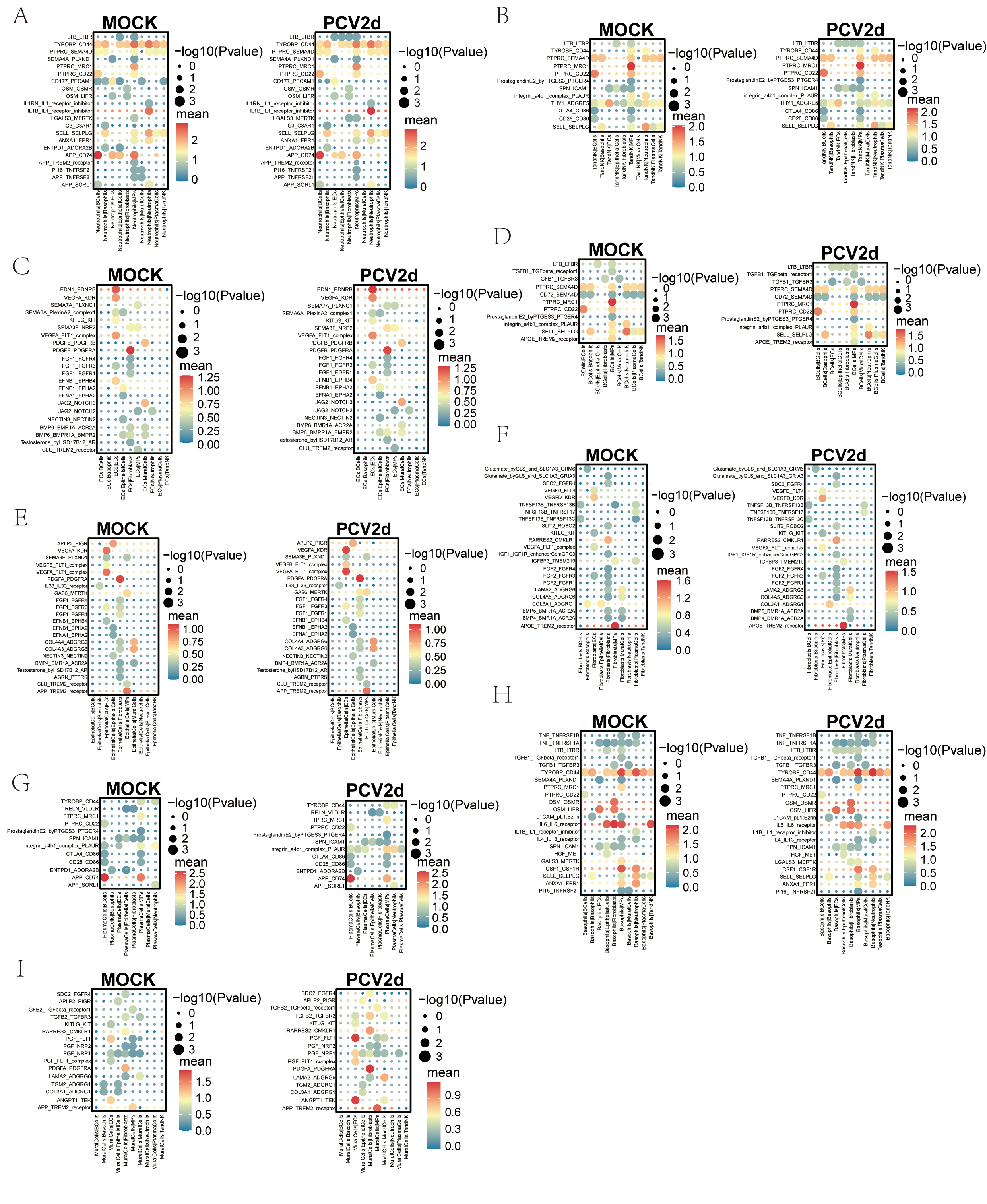

Supplement: Supplementary Figure S4 — Cellular communication of the remaining nine subpopulations of cells in the MOCK and PCV2d groups, respectively. (A) Communication pathways of Neutrophils subpopulations with other subpopulations under the two treatment groups, darker colors represent stronger mutualisms. (B) Communication pathways of TandNK subpopulations with other subpopulations under the two treatment groups, darker colors represent stronger mutualisms. (C) Communication pathways of Endothelial subpopulations with other subpopulations under the two treatment groups, darker colors represent stronger mutualisms. (D) Communication pathways of BCells subpopulations with other subpopulations under the two treatment groups, darker colors represent stronger mutualisms. (E) Communication pathways of Epithelial cell subpopulations with other subpopulations under the two treatment groups, darker colors represent stronger mutualisms. (F) Communication pathways of Fibroblast subpopulations with other subpopulations under the two treatment groups, darker colors represent stronger mutualisms. (G) Communication pathways of Plasma cell subpopulations with other subpopulations under the two treatment groups, darker colors represent stronger mutualisms. (H) Communication pathways of Basophils subpopulations with other subpopulations under the two treatment groups, darker colors represent stronger mutualisms. (I) Communication pathways of Mural cell subpopulations with other subpopulations under the two treatment groups, darker colors represent stronger mutualisms. [file Image_4.tif]
